# Selectively superior production of docosahexaenoic acid in *Schizochytrium* sp. through engineering the fatty acid biosynthetic pathways

**DOI:** 10.1186/s13068-024-02524-2

**Published:** 2024-06-03

**Authors:** Yana Liu, Xiao Han, Zongcheng Chen, Yihan Yan, Zhi Chen

**Affiliations:** 1 State Key Laboratory of Animal Biotech Breeding, College of Biological Sciences, China Agricultural University, Beijing, 100193 China; 2 College of Life Sciences, Qingdao Agricultural University, Qingdao, 266109 China

**Keywords:** *Schizochytrium* sp., Docosahexaenoic acid, Fatty acid synthase, PUFA synthase, Phosphopantetheinyl transferase

## Abstract

**Background:**

*Schizochytrium* sp. is commercially used for production of docosahexaenoic acid (DHA). *Schizochytrium* sp. utilizes the polyketide synthase complex (PKS) and a single type I fatty acid synthase (FAS) to synthesize polyunsaturated fatty acids and saturated fatty acids, respectively. The acyl carrier protein (ACP) domains of FAS or PKS are used to load acyl groups during fatty acids biosynthesis. Phosphopantetheinyl transferase (PPTase) transfers the pantetheine moiety from Coenzyme A to the conserved serine residue of an inactive ACP domain to produce its active form.

**Results:**

In this study, in order to improve production and content of DHA, we decreased the expression of *fas*, strengthened the expression of the PKS pathway, and enhanced the supply of active ACP in *Schizochytrium* sp. ATCC20888. Weakening the expression of *fas* or disruption of *orfA* both led to growth defect and reduction of lipid yields in the resulting strains WFAS and DPKSA, indicating that both FAS and PKS were indispensable for growth and lipid accumulation. Although WFAS had a higher DHA content in total fatty acids than the wild-type strain (WT), its growth defect and low DHA yield hinders its use for DHA production. Overexpression of the *orfAB*, *orfC*, *orfC*-*DH* (truncated *orfC*), or *ppt* promoted DHA and lipid production, respectively. The yields and contents of DHA were further increased by combined overexpression of these genes. Highest values of DHA yield (7.2 g/L) and DHA content (40.6%) were achieved in a recombinant OPKSABC-PPT, ⁓56.5% and 15.3% higher than the WT values, respectively.

**Conclusions:**

This study demonstrates that genetic engineering of the fatty acid biosynthetic pathways provides a new strategy to enhance DHA production in *Schizochytrium*.

**Supplementary Information:**

The online version contains supplementary material available at 10.1186/s13068-024-02524-2.

## Background

Omega-3 polyunsaturated fatty acids (PUFAs), such as DHA (22:6 ω-3) and eicosapentaenoic acid (EPA, 20:5 ω-3), play important roles in promoting development of nervous system and visual system and preventing cardiovascular and cerebrovascular diseases, diabetes, and cancers, and are widely used in food additives and pharmaceutical industry [1–3].

**Fig. 1:**
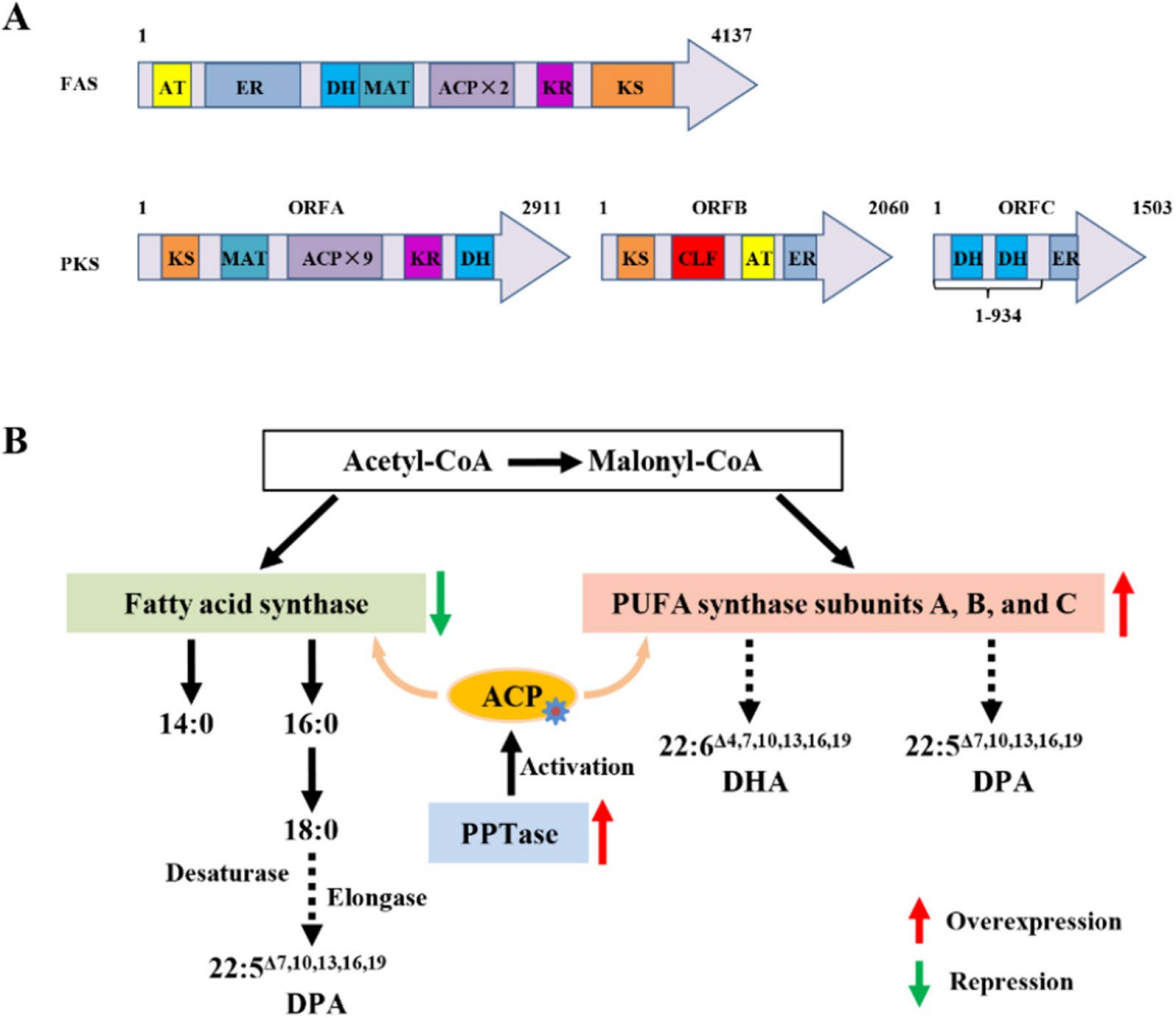
Schematic diagram of the fatty acid biosynthetic pathways in *Schizochytrium* sp. ATCC20888. **A** Domain organization in polyketide synthase (PKS) and fatty acid synthase (FAS) in *Schizochytrium* sp. *ACP*, acyl carrier protein; *AT,* acyltransferase; *CLF*, chain length factor; *DH*, dehydratase; *ER,* enoyl-ACP reductase; *MAT,* malonyl-CoA: *ACP* acyl transferase; *KR*, 3-ketoacyl-ACP reductase; *KS,* 3-ketoacyl-ACP synthase. **B** Genetic engineering of the fatty acid biosynthetic pathways for improved production of DHA in *Schizochytrium* sp. Gene overexpression is shown in red arrow and gene repression in green arrow

In recent years, *Schizochytrium* sp. of thraustochytrids has attracted wide interests for its ability to accumulate significant amounts of total lipids rich in DHA [4]. Fatty acid biosynthesis in *Schizochytrium* sp. involves two different pathways that work independently. A single type I fatty acid synthase is responsible for saturated fatty acids (SFAs) biosynthesis (Fig. 1A)*.* The polyketide synthase complex (PKS or PUFA synthase) is responsible for biosynthesis of DHA and docosapentaenoic acid (DPA, 22:5 ω-6) [5–7]. FAS contains one acyltransferase (AT), one enoyl reductase (ER), one dehydratase (DH), one malonyl-CoA: ACP acyl transferase (MAT), two acyl carrier protein (ACP), one 3-ketoacyl-ACP reductase (KR), and one 3-ketoacyl-ACP synthase (KS) domains. FAS catalyzes a set of iterative reactions where two carbons are added at each cycle, eventually producing a saturated C16 [6, 8]. The PUFA synthase complex in *Schizochytrium* sp. is made of 3 subunits (ORFA, ORFB, and ORFC; also known as PFA1, PFA2, and PFA3), each also harboring different multi-domains (Fig. 1A). ORFA consists of one KS, one MAT, nine ACP, one KR, and one DH domains. ORFB consists of one KS, one chain length factor (CLF), one AT, and one ER domains. ORFC consists of two DH and one ER domains [4, 5, 8]. PUFA synthase also catalyzes a set of iterative reactions that add two carbons at each cycle, while the specific mechanism how these two carbons are processed remains unclear. To be functional, PUFA synthase complex or FAS require a phosphopantetheinyl transferase (PPTase) to activate their ACPs through transferring the pantetheine moiety from Coenzyme A to the serine residues of ACPs [6, 9]. The number of tandemly repeated ACP domains in PUFA synthase has a positive correlation with its PUFA productivity [10, 11].

Although there is sparse detailed knowledge of the thraustochytrids PUFA synthases, various domains of the PUFA synthases are potential targets for enhancement of PUFAs productivity. Heterologous expression of *orfABC* genes of *Schizochytrium* sp. in *E. coli* or *Yarrowia lipolytica* led to accumulation of long-chain PUFAs in resulting recombinants [6, 12]. Overexpression of the KS domain of ORFA subunit from *Thraustochytrium* sp. ATCC26185 in *E. coli* improved production of SFAs effectively, while overexpression of the ORFB KS domain led to a higher ratio of unsaturated fatty acids to SFAs [13]. Disruption of the CLF domain of ORFB or the second DH domain of ORFC in *Schizochytrium* sp. significantly decreased PUFAs yield but slightly increased SFAs yield [14]. Overexpression of malonyl-CoA: ACP transacylase in *Schizochytrium* sp. increased total lipids and DHA yields by 39.60% and 81.50% in fed-batch fermentation, respectively [15]. Overexpression of an endogenous phosphopantetheine transferase gene (*ppt_a*) in *Aurantiochytrium* sp. enhanced yield and proportion of DHA by 35.5% and 17.6%, respectively [9]. Overexpression of the ORFC DH domain in *Schizochytrium limacinum* SR21 increased DHA and DPA contents by 9.8% and 14.8%, respectively, while overexpression of the ORFC ER domain increased SFAs content and decreased DHA and DPA contents [16]. *Aurantiochytrium* sp. SD116 contains two copies of *fas*, and DHA content in total fatty acids (TFAs) was increased from 41 to 61% through deleting a copy of *fas* and overexpression of acetyl-CoA carboxylase and diacylglycerol acyltransferase [17].

*Schizochytrium* sp. has become an emerging new model for biotechnological applications with improvements of the transformation techniques and genetic tools. In this study, we decreased the expression of *fas*, strengthened the expression of PPTase and three subunits of the PUFA synthase complex (ORFA, ORFB, and ORFC) in *Schizochytrium* sp. ATCC20888 (Fig. 1B). DHA yield and DHA content of TFAs in a recombinant OPKSABC-PPT reached 7.2 g/L and 40.6%, which were increased by 56.5% and 15.3% compared to WT, respectively. The investigation indicates that genetic engineering of the fatty acid biosynthetic pathways is an efficient way to improve DHA production in thraustochytrids.

## Results

### Decreased expression of *fas* increased DHA content but hindered growth

**Fig. 2:**
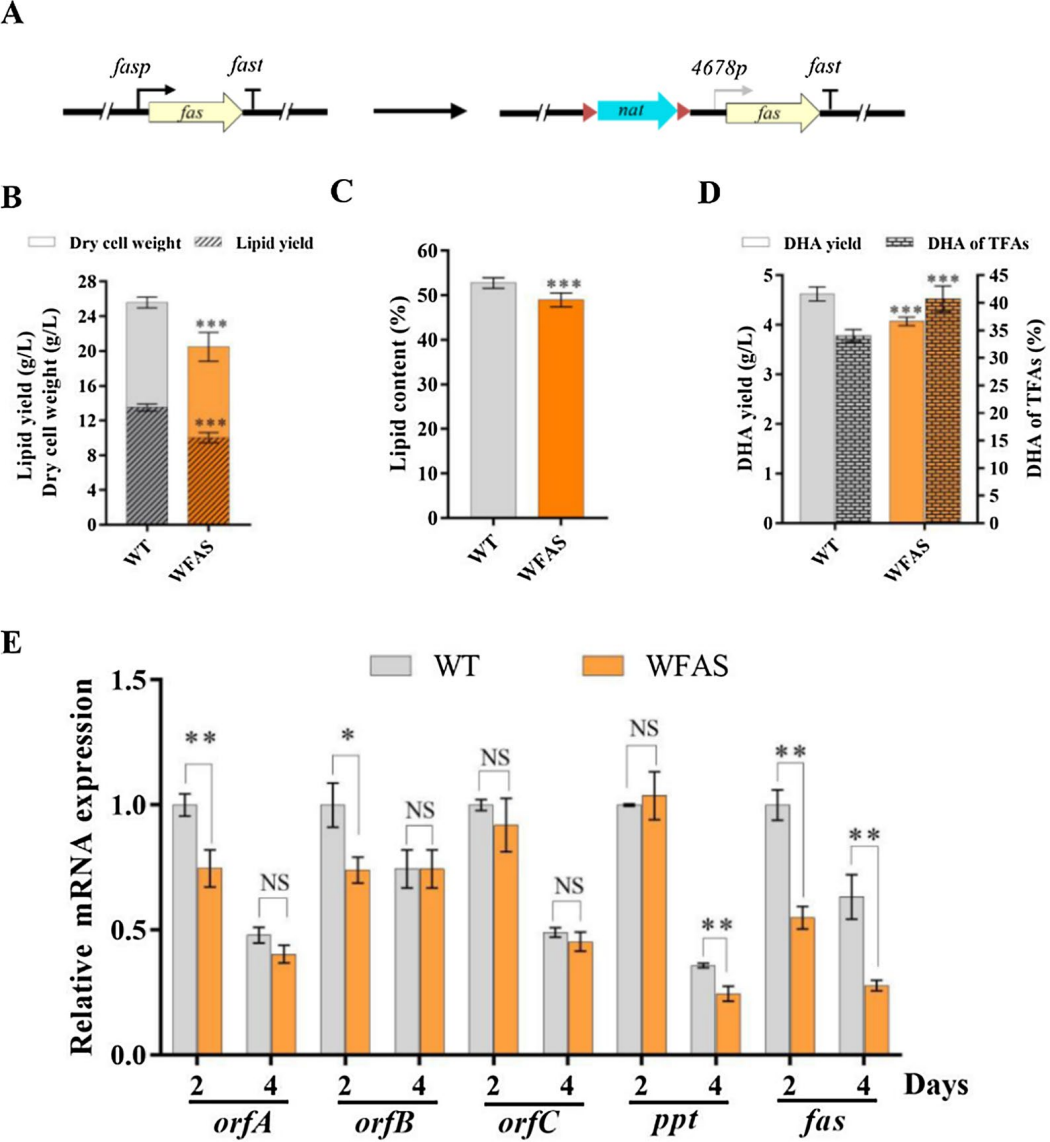
Effects on growth, lipid accumulation, and DHA production by decreasing expression of *fas*. **A** Schematic illustration of WFAS construction. Gray arrow: weak promoter. **B** Dry cell weight (DCW; g/L) and lipid yield (g/L). **C** Lipid content (% DCW). **D** DHA yield (g/L) and DHA content (% TFA). Values are mean ± SD from three replicate flasks grown in fermentation medium for 120 h. **E** RT-qPCR analysis of transcription levels of the fatty acid biosynthetic genes in WFAS and WT. *p < 0.05, **p < 0.01, ***p < 0.001; NS, not significant (Student *t* test)

Saturated fatty acids (C14:0 and C16:0) of *Schizochytrium* are synthesized by FAS. *Schizochytrium* has one *fas* gene and deletion of the only *fas* gene may be lethal. In order to increase the proportion of PUFAs (especially DHA) in TFAs, we decreased expression of the *fas* gene by displacing its promoter with a weak promoter *4678p* through homologous recombination (Fig. 2A, S1). The expression level of *4678p* in WT was much lower than that of *fas*, especially at the time point (72 h) when lipid is rapidly accumulated (Fig. S1A). Weakening the expression of *fas* hindered growth, and the growth defect could be recovered by supplementation with total lipids from WT to the medium (Fig. 2B, S2).

Lipid body staining intensity was weaker for WFAS than for WT (Fig. S3). Compared to WT, the biomass and lipid yield of the *fas*-weakened strain WFAS were decreased by 19.6% and 24.2% (Fig. 2B, C). Although the DHA percentage in TFAs of WFAS (40.7%) was higher than that of WT (34.1%), the DHA yield was decreased by 10.9% due to the decreased growth (Fig. 2D). RT-qPCR analysis revealed that the transcription levels of *fas* in WFAS were 55.0% and 44.0% of those in WT at 2 and 4 d. And the transcription levels of the PUFA synthase genes (*orfA*, *orfB*, and *orfC*) and the phosphopantetheine transferase gene (*ppt*) in WFAS were similar to or slightly lower than those in WT, which was consistent with the lipid yields of WT and WFAS (Fig. 2E). The findings indicate that decreasing the FAS pathway of *Schizochytrium* increases DHA content but impairs cell growth. Therefore, it is inadvisable to completely or partially disrupt the FAS pathway to improve the DHA content in *Schizochytrium* sp.

### Insertion inactivation of *orfA* interrupted DHA synthesis

**Fig. 3:**
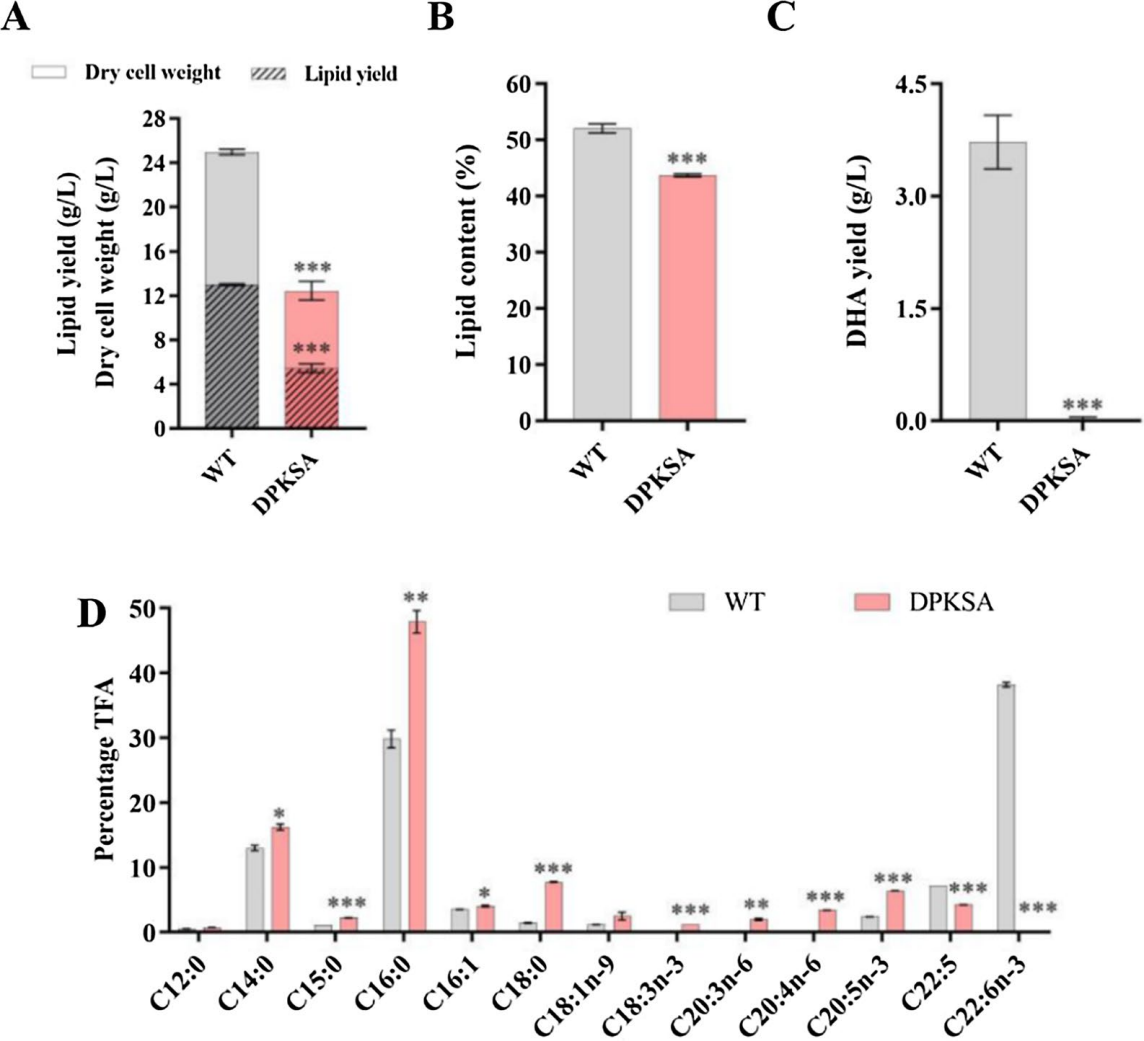
Growth, lipid accumulation, and DHA production of WT and DPKSA. **A** DCW and lipid yield (g/L). **B** Lipid content (% DCW). **C** DHA yield (g/L). **D** Fatty acid composition (% TFA) in WT and DPKSA cultured for 120 h. *p < 0.05, **p < 0.01, ***p < 0.001 (Student *t* test)

Decreasing the FAS pathway of *Schizochytrium* increases DHA content in TFAs but impairs growth and DHA yield, therefore, it is not wise to use WFAS for DHA production. *Schizochytrium* sp. utilizes the PKS pathway to synthesize PUFAs. In order to determine the function of PUFA synthase in fatty acid biosynthesis, we disrupted the *orfA* gene by inserting the selective marker *bleomycin* into its coding region (Fig. S4). Disruption of *orfA* greatly impaired growth, and supplementation with total lipids from WT also restored its growth to the WT level (Fig. 3A, S2). The *orfA*-inactivated mutant DPKSA produced much less lipid than WT (Fig. 3A, B, S3; Table 1).

**Table 1:** Fermentation characteristics of *Schizochytrium* sp. strains

Strain	DCW (g/L)	Lipid yield (g/L)	Lipid content (%)	DHA yield (g/L)
WT	25.5 ± 0.6^b^	13.2 ± 0.3^d^	51.8^d^	4.6 ± 0.3^b^
DPKSA	12.4 ± 0.8^d^	5.4 ± 0.4^f^	43.5^f^	0.03 ± 0.03^d^
WFAS	20.5 ± 1.6^c^	10.0 ± 0.6^e^	48.8^e^	4.1 ± 0.1^c^
OPKSAB	25.8 ± 0.5^b^	16.0 ± 0.2^c^	62.0^c^	6.3 ± 0.3^a^
OPKSC	26.8 ± 0.1^a^	16.9 ± 0.3^b^	63.1^bc^	6.4 ± 0.1^a^
OPKSCDH	25.9 ± 0.3^b^	16.1 ± 0.3^c^	62.2^c^	6.3 ± 0.3^a^
OPPT	25.9 ± 0.3^b^	16.6 ± 0.7^bc^	64.1^bc^	6.3 ± 0.3^a^
OPKSAB-PPT	26.1 ± 0.2^b^	16.8 ± 0.4^b^	64.4^bc^	6.4 ± 0.3^a^
OPKSC-PPT	26.4 ± 1.0^ab^	17.1 ± 1.0^b^	64.8^bc^	6.8 ± 0.3^a^
OPKSCDH-PPT	26.3 ± 0.4^ab^	17.4 ± 0.3^b^	66.2^b^	6.6 ± 0.1^a^
OPKSABCDH	26.6 ± 0.8^ab^	16.6 ± 0.6^bc^	62.4^c^	6.6 ± 0.4^a^
OPKSABCDH-PPT	26.1 ± 0.2^b^	17.3 ± 0.9^b^	66.3^b^	6.8 ± 0.7^a^
OPKSABC-PPT	26.1 ± 0.6^b^	18.7 ± 0.6^a^	71.6^a^	7.2 ± 0.5^a^

Data were analyzed by one-way ANOVA and Duncan’s multiple range test, using SPSS V. 23.0

Differing lowercase letters indicate significant difference (*p* < 0.05) between values

DPKSA produced almost no DHA and less DPA (Fig. 3, S5), indicating that the PKS pathway is responsible for de novo synthesis of DHA and DPA, which is in accord with the previous studies [14]. The DPA percentage in TFAs of DPKSA (4.25%) was lower than WT (7.15%), suggesting that DPA synthesis depends on both FAS and PKS pathways. The proportions of C14:0 and C16:0 in TFAs of DPKSA were significantly increased. Besides, the proportions of C18:0, 18:3n–3, 20:3n–6, 20:4n–6, and EPA were also dramatically increased (Fig. 3D, S5), implying that there is a partial desaturase/elongase pathway in *Schizochytrium*. The silent pathway seemed to be activated in DPKSA, probably compensating for the loss of PUFAs (DHA and DPA) synthesis. Therefore, PUFA synthase plays an indispensable role in DHA synthesis and growth of *Schizochytrium* sp., while EPA is synthesized by FAS and the desaturase/elongase pathway.

Decreased expression of the PKS or FAS pathway impaired growth, which could be restored by addition of WT total lipids. Thin layer chromatography (TLC) analysis indicated that DPKSA and WFAS produced less TAG than WT (Fig. S6A). The PUFAs contents in TAG or phospholipids of DPKSA and WFAS resembled their PUFAs content of TFAs (Fig. S6B–E). The findings suggest that impaired growth of DPKSA and WFAS might be due to their imbalanced fatty acid composition of cell membranes, which affects membrane fluidity and stability, thereby impairing normal growth of cells. Addition of WT total lipids to the mutants exogenously compensated the corresponding fatty acid components and restored growth defect.

### Overexpression of PKS or PPT increased DHA and lipid production

**Fig. 4:**
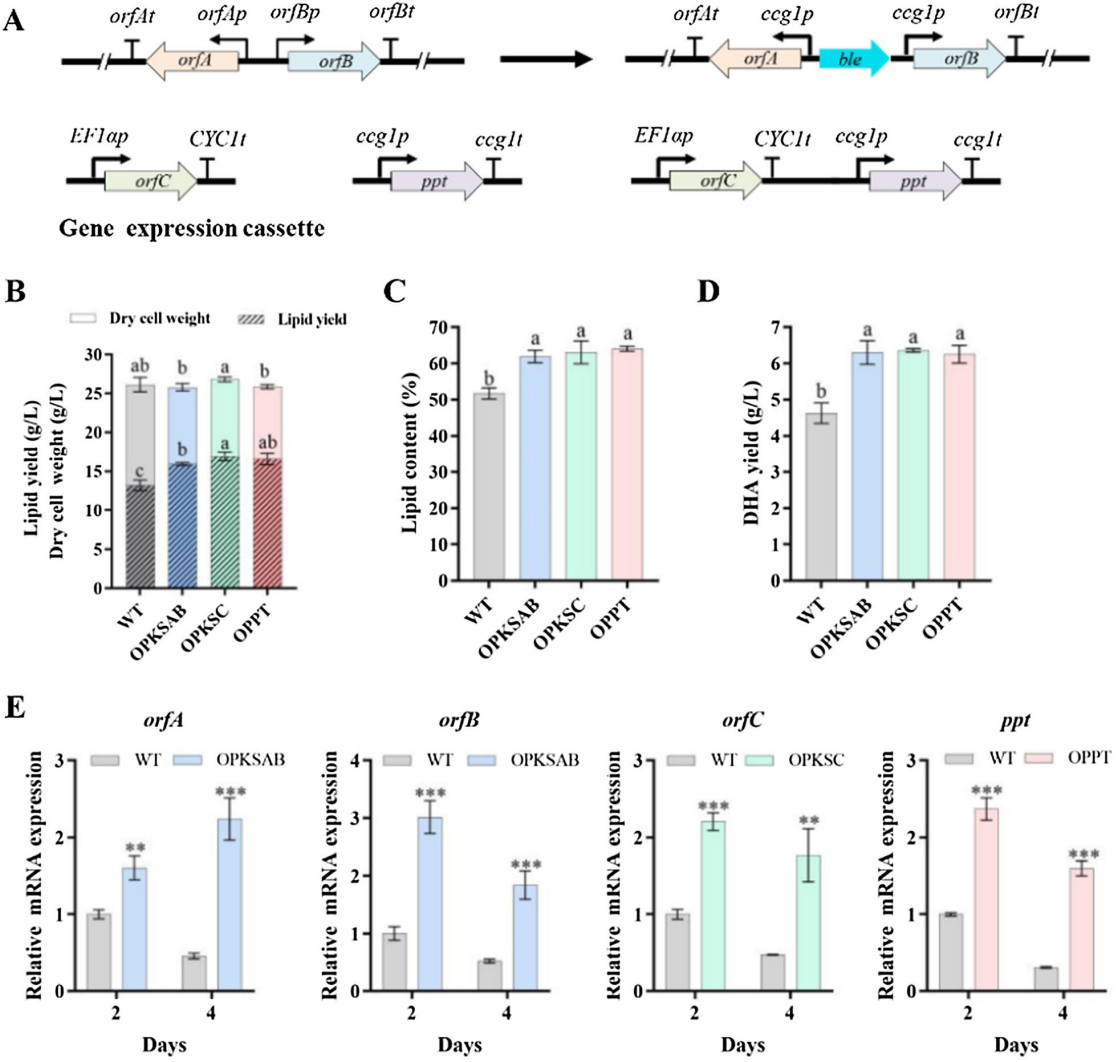
Effects on growth, lipid accumulation, and DHA production by overexpression of *pks* and *ppt* genes. **A** Schematic illustration of construction of overexpression strains. Bold black arrow: strong promoter. **B** DCW and lipid yield (g/L). **C** Lipid content (% DCW). **D** DHA yield (g/L). **E** RT-qPCR analysis of the overexpressed genes in OPPT, OPKSAB, and OPKSC. Statistical analyses were performed using one-way ANOVAs and Duncan’s multiple range tests. Different lowercase letters in each bar indicate a significant difference (*p* < 0.05) between values

In order to promote the metabolic flow from SFAs synthesis towards DHA synthesis, we overexpressed the three genes *(orfA*, *orfB*, and *orfC*) encoding the PUFA synthase complex. *orfA* shares a bidirectional promoter region with *orfB*, since both genes are too big to handle easily in plasmids, we in situ co-overexpressed *orfA* and *orfB* through substituting their promoter regions with two *ccg1* promoters in opposite directions by homologous recombination (Fig. 4A, S7). And *orf*C overexpression strain was constructed by introduction of an extra copy of *orfC* promoted by strong promoter *EF1αp* to chromosome (Fig. 4A). The large multifunctional ORFA of the PKS complex harbors nine multi-tandem ACP domains, and PPTase transfers the pantetheine moiety from Coenzyme A to the conserved serine residue of an inactive ACP domain to produce its active form. Therefore, *ppt* was overexpressed by introduction of an extra copy promoted by *ccg1* promoter (Fig. 4A).

The dry cell weights of these overexpression mutants (OPKSAB, OPKSC, and OPPT) were similar to that of WT (Fig. 4B). Relative to WT value (51.8%), lipid contents were much higher for OPKSAB (62.0%), OPKSC (63.1%), and OPPT (64.1%). The yields of lipid and DHA in OPKSAB (16.0 and 6.3 g/L), OPKSC (16.9 and 6.4 g/L), and OPPT (16.6 and 6.3 g/L) were significantly higher than those of WT (13.2 and 4.6 g/L) (Fig. 4B–D; Table 1). Compared to WT, the transcription levels of *ppt*, *orfA*, *orfB*, and *orfC* were significantly increased (> 1.5 fold) in respective overexpression strains, indicating that the genes were successfully overexpressed (Fig. 4E). The results indicate that overexpression of ORFAB, ORFC, and PPT enhanced lipid and DHA yields of *Schizochytrium* sp. The results are consistent with the previous studies that overexpression of PPT or ORFC enhanced PUFAs production of *Aurantiochytrium* or *S. limacinum* SR21 [9, 16].

### Combinatorial genetic engineering for enhanced DHA and lipid production

**Fig. 5:**
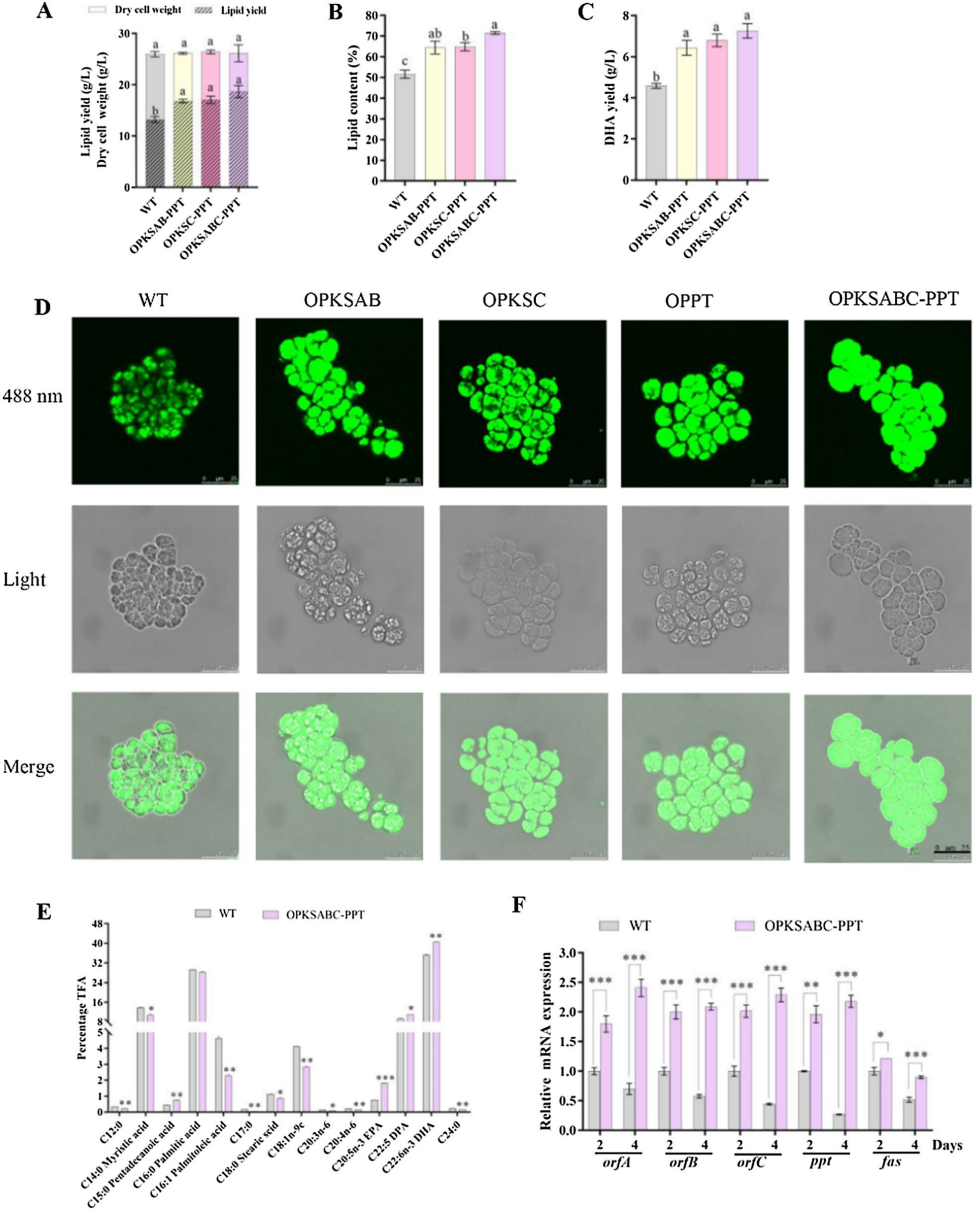
Growth, lipid accumulation, DHA production, and gene expression of WT and combined overexpression strains of *pks* and *ppt* genes. **A** DCW and lipid yield (g/L). **B** Lipid content (% DCW). **C** DHA yield (g/L). The different lowercase letters in each bar indicate a significant difference (*p* < 0.05) between values. **D** Confocal microscopy images of Nile red-stained cells grown in fermentation medium for 72 h. Fluorescence intensity was measured using an excitation wavelength of 488 nm (staining color: green). Bar, 25 μm. **E** Fatty acid composition (% TFA) in WT and OPKSABC-PPT. (F) RT-qPCR analysis of transcription levels of fatty acid biosynthetic genes in WT and OPKSABC-PPT. *p < 0.05, **p < 0.01, ***p < 0.001 (Student *t* test)

In order to further improve DHA yield, the expression cassettes of *ppt* and *ppt*-*orfC* were transformed into OPKSAB or WT (Fig. 4A). Compared to WT, OPKSAB, OPKSC, and OPPT, the Nile red-based fluorescences were improved in co-overexpression strains OPKSAB-PPT, OPKSC-PPT and OPKSABC-PPT (Fig. S8). Co-expression of these genes did not affect DCWs (Fig. 5A), but promoted DHA and lipid production. The highest lipid and DHA yields (18.7 and 7.2 g/L) were achieved in OPKSABC-PPT, increased by 41.7% and 56.5% relative to WT values (Fig. 5A-C). Lipid body-staining of OPKSABC-PPT were stronger than WT, OPKSAB, OPKSC, and OPPT (Fig. 5D).

To determine whether overexpression of the PKS complex selectively increases DHA production, we analyzed the fatty acid composition of total lipids from OPKSABC-PPT and WT. The proportions of TFAs corresponding to DHA (40.6%) and DPA (11.0%) were higher in OPKSABC-PPT than in those of WT (DHA 35.2%; DPA 9.3%); accordingly, the proportions represented by myristic acid (C14:0) and palmitoleic acid (C16:1) were reduced in OPKSABC-PPT (Fig. 5E). The findings suggest that overexpression of PKS and PPT promotes metabolic fluxes to the PKS pathway, resulting in enhanced DHA accumulation. Although the proportions of TFAs represented by DHA and DPA were increased, the improved extent is quite small. Therefore, the transcriptional levels of key genes related to DHA and SFAs synthesis were investigated in OPKSABC-PPT and WT by RT-qPCR. The transcription levels of *ppt, orfA*, *orfB*, and *orfC* were greatly increased (> 1.5 fold) in OPKSABC-PPT compared with those in WT (Fig. 5F). However, the expression level of *fas* was also evidently increased in OPKSABC-PPT. The RT-qPCR results are consistent with enhanced DHA and lipid yields of OPKSABC-PPT. How *fas* is upregulated in OPKSABC-PPT needs further investigations.

**Fig. 6:**
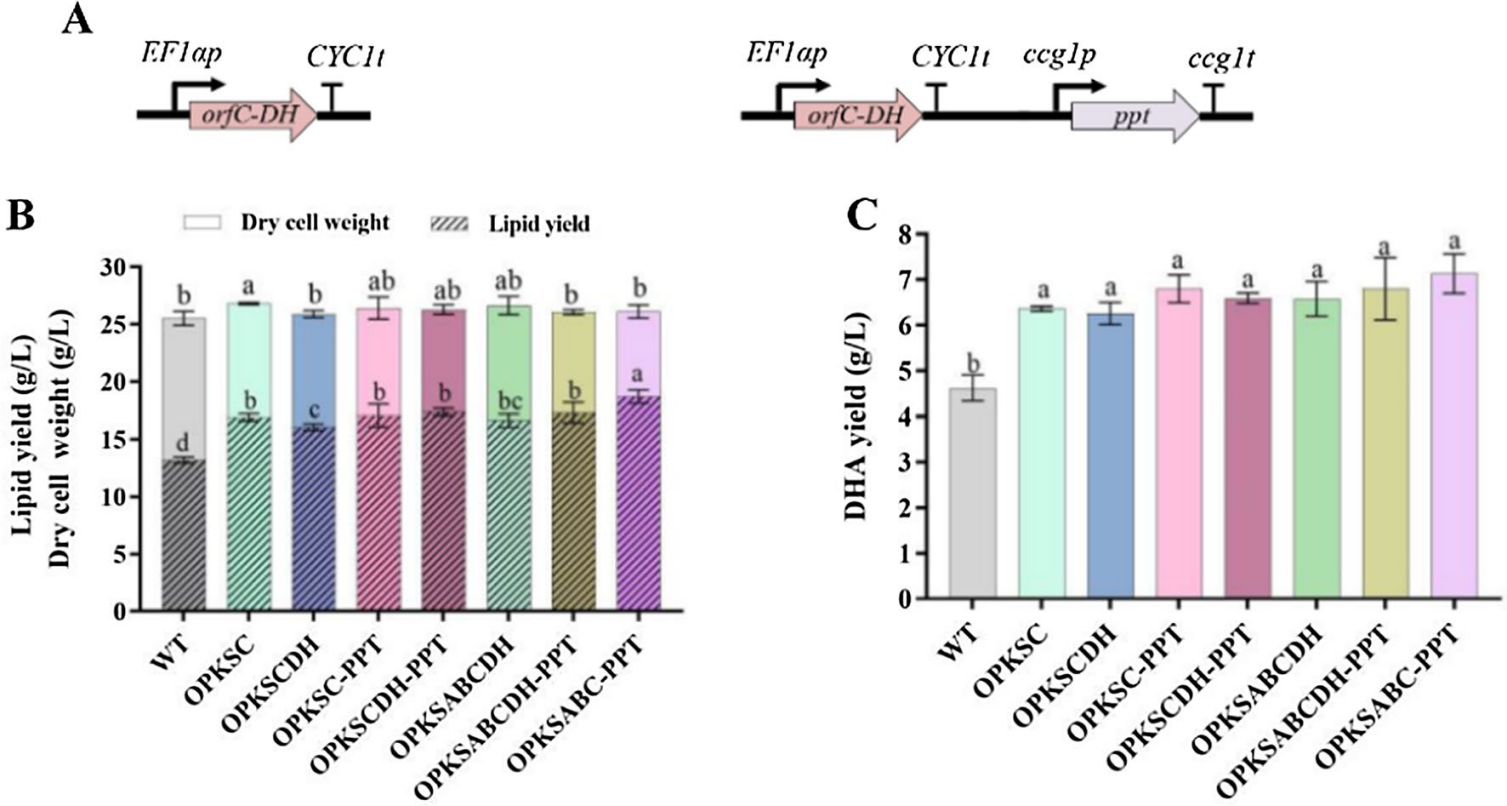
Fermentation of WT and combined overexpression strains of truncated *orfC*. **A** Schematic illustration of construction of overexpression strains. Bold black arrow: strong promoter. **B** DCW and lipid yield (g/L). **C** DHA yield (g/L)

ORFC contains two DH domains at N terminus and one ER domain at C terminus. Shi et al. [16] has shown that overexpression of DH domains of ORFC specifically promoted synthesis of PUFAs, while overexpression of ER domain promoted SFAs synthesis of *S. limacinum* SR21. Therefore, *orfC-DH* (truncated *orfC*, in which DNA encoding the ER domain was deleted) was overexpressed or co-overexpressed with *ppt* in WT or OPKSAB to promote DHA production (Fig. 6A). Overexpression of ORFC-DH did not affect dry cell weights of WT or OPKSAB. Compared to WT (13.2 g/L), lipid yields were significantly improved in OPKSCDH (16.1 g/L) and OPKSABCDH (16.6 g/L), especially in OPKSCDH-PPT (17.4 g/L) and OPKSABCDH-PPT (17.3 g/L) (Fig. 6B). However, their DHA yields were not selectively promoted as expected, even slightly lower than those of OPKSC-PPT and OPKSABC-PPT (Fig. 6C).

### Time course fermentation performance of the engineered strain OPKSABC-PPT

**Fig. 7:**
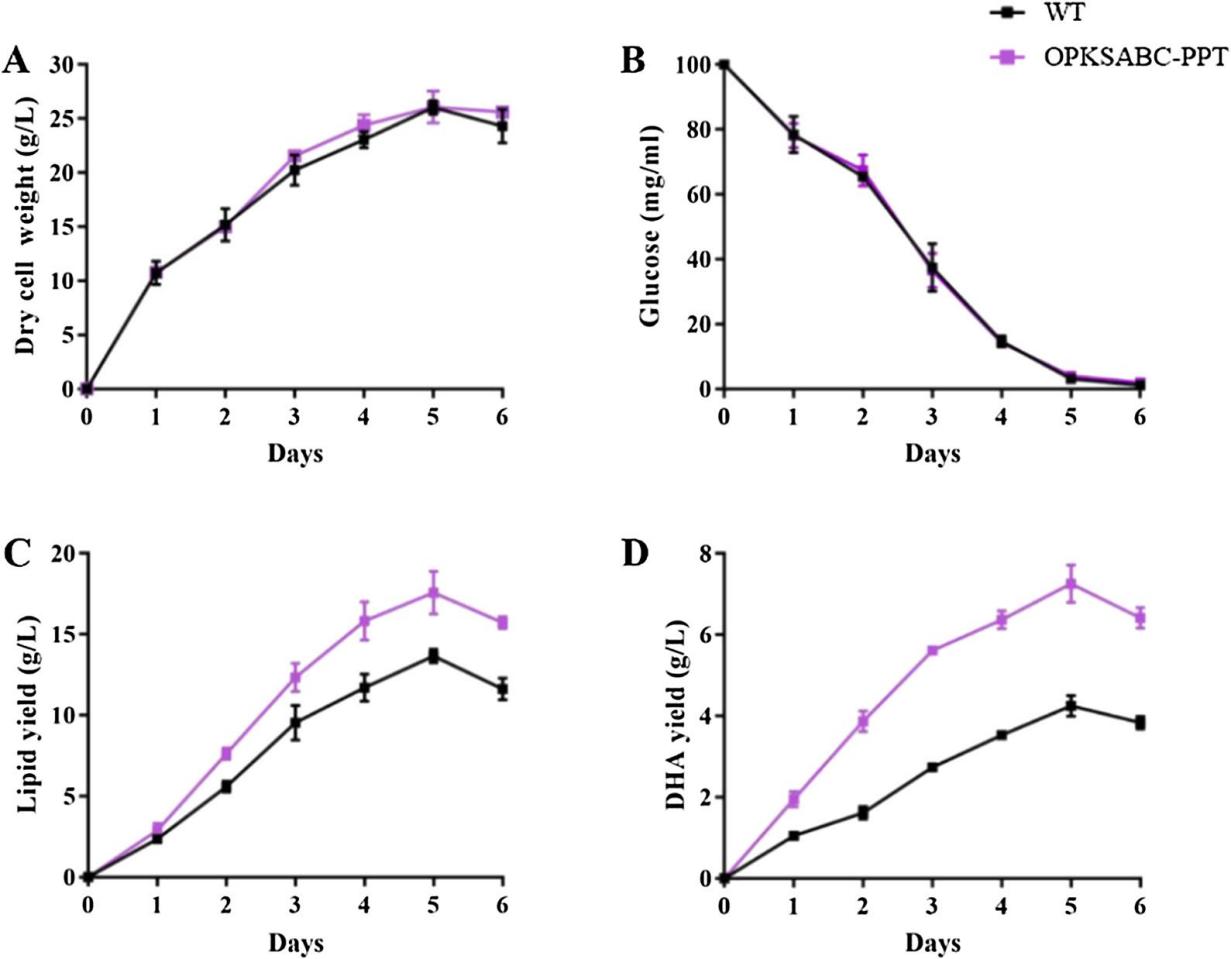
Fermentation curves of WT and OPKSABC-PPT. **A** DCW (g/L). **B** Glucose consumption (g/L). **C** Lipid yield (g/L). **D** DHA yield (g/L)

The highest DHA yield was achieved in OPKSABC-PPT. Therefore, we determined glucose utilization, biomass, lipid yield, and DHA yield profiles of OPKSABC-PPT and WT. OPKSABC-PPT had similar glucose utilization and biomass curves to WT (Fig. 7A, B). The DCWs, lipid yields, and DHA yields of both strains reached their maximum values on day 5 when glucose was almost consumed up, and deceased with further cultivation. The yields of lipid and DHA in OPKSABC-PPT on day 5 were ⁓ 41.7% and 56.5% higher than those in WT (Fig. 7C, D). The high DHA yield of OPKSABC-PPT highlights it potential for industrial application.

## Discussion

In this study, DHA yield and DHA content in TFAs were increased through co-overexpression of the three subunits of PUFA synthase and PPTase in *Schizochytrium* sp. ATCC20888. The DHA yield of OPKSABC-PPT reached 7.2 g/L, accounting for 40.6% of TFAs, which are about ⁓56.5% and 15.3% higher than the WT values.

Various metabolic engineering strategies have been exploited to promote DHA accumulation of thraustochytrids. *Aurantiochytrium* sp. SD116 contains two copies of *fas*, and DHA content was increased from 41 to 52% by deleting a copy of *fas* without affecting growth, and was further increased to 61% through overexpression of *acc* and *dgaT* in ∆fas [17]. *Schizochytrium* sp. ATCC20888 contains only one copy of *fas*, and weakening the expression of *fas* successfully increased DHA content but seriously impaired growth, indicating that FAS is essential in *Schizochytrium* sp. An engineered yeast comprising EPA at 56.6% of TFAs and less than 5% SFAs has been constructed in *Y. lipolytica*, in which the EPA content in TAG resembled the EPA content of TFAs, while the EPA content in phospholipids was only about 22% of TFAs [18]. In this study, we found that either decreasing the expression of *fas* or strengthening the expression of *orfABC* increased DHA proportion in TFAs of *Schizochytrium* sp. ATCC20888, but in very small scales. In *Thraustochytrium* sp., very long chain polyunsaturated fatty acids (VLCPUFAs) accumulated in TAG are channeled from phosphatidylcholine, almost exclusively located at its *sn*-2 position, while SFAs are preferentially located at its *sn*-1/3 positions [19]. Although phosphatidylcholine may have either one VLCPUFA at its *sn*-2 position or two VLCPUFAs, lipid is mainly accumulated in the form of TAG in thraustochytrids. That is probably the reason why we couldn’t greatly improve the DHA proportion in TFAs in *Schizochytrium* sp. Thus, introduction of heterologous glycerol-3-phosphate acyltransferase, lysophosphatidic acid acyltransferase, and acyl-CoA: diacylglycerol acyltransferase with preference for VLCPUFA to thraustochytrids might promote DHA accumulation in TAG and enhance DHA content.

*Schizochytrium* sp. de novo synthesizes DHA via the PUFA synthase. Previous studies demonstrated the importance of ORFA and ORFB of the PUFA synthase in DHA production and cell growth in *Schizochytrium* spp. [7, 20]. In this study, overexpression of ORFA and ORFB significantly enhanced DHA accumulaiton without affecting growth. Shi’s investigation indicated that overexpression of the DH domains and ER domain of ORFC specifically promoted synthesis of PUFAs and SFAs in *S. limacinum* SR21, respectively [16]. However, overexpression of the DH domains of ORFC (ORFC-DH) in *Schizochytrium* sp. ATCC20888 displayed a slightly lower DHA yield than overexpression of the entire ORFC, especially when co-expressed with ORFAB and PPTase. So far, lack of crystal structures of the PUFA synthases limits our understanding of the processes catalyzed by these enzymes. Mammalian and fungal FAS have been demonstrated to be a 540-kDa homodimer and a 2.6-megadalton α6β6 heterododecameric complex [21, 22]. The three different subunits of the PUFA synthase may form similar giant multidomain complex like FAS, whereas the exact architecture remains to be discovered. Truncated ORFC may alter protein conformation of the PUFA synthase complex, which is unbeneficial to PUFAs synthesis. PPTase activates the ACP domains of PUFA synthase, and overexpression of PPTase significantly increased DHA and PUFA proportions in *Aurantiochytrium* sp. SD116 and *Schizochytrium* sp. HX-308 [9, 23]. Overexpression of PPTase also greatly increased DHA production and DHA proportion in *Schizochytrium* sp. ATCC20888, especially when co-expressed with ORFA, ORFB, ORFC, indicating that PPTase plays critical roles in DHA biosynthesis.

Thraustochytrids exploit two independent pathways (PKS and FAS) for synthesis of fatty acids, while the underlying mechanism remains to be elucidated. Mutant devoid of PUFA synthase activity displays growth defect phenotype and is auxotroph for PUFAs [7]. Here, we found that either disruption of *orfA* or deceasing expression of *fas* in *Schizochytrium* sp. both led to growth defect phenotype, which could be recovered by addition of total lipids from WT to culture medium, indicating that both pathways are essential for growth. Phospholipids are the principal components of cell membrane, and proportions of saturated and unsaturated fatty acids will affect fluidity, stability, and permeability of the membrane, thus affecting growth of cells [24]. DPKSA had a lower PUFAs proportion of phospholipids, while WFAS had a lower SFAs proportion of phospholipids (Fig. S6), thus, their imbalanced proportions of phospholipids are probably the reason of growth defect. The fatty acid profile of DPKSA demonstrated the existence of a silent desaturase/elongase pathway in *Schizochytrium*, which is activated to compensate for the loss of PUFAs synthesis in DPKSA.

Disruption of *orfA* led to loss of DHA synthesis and increased production of 18:3n-3, 20:3n-6, 20:4n-6, and EPA (Fig. 3D). Decreasing the expression of *fas* also slightly decreased the expression of *orfABC* in WFAS, while overexpression of *orfABC* genes was accompanied by a slight increase in *fas* expression in OPKSABC-PPT (Fig. 2E, 5F), suggesting that the synthesis of SFAs and PUFAs in *Schizochytrium* sp. is tightly controlled to maintain membrane homeostasis. In baker’s yeast, Mga2 senses the molecular lipid-packing density in endoplasmic reticulum membrane and adjusts membrane lipid saturation through regulating transcription of the fatty acid Δ9-desaturase Ole1 [25, 26]. Although *Schizochytrium* sp. possesses partial desaturase/elongase system, it synthesizes unsaturated fatty acids mainly via PUFA synthase. In our previous studies, we have identified zinc finger protein LipR and bZIP transcription factor FabR which both simultaneously represses the transcription of fatty acids synthase and PUFA synthase genes in *Schizochytrium* sp. [27, 28]. Therefore, systematical screening and validation of the regulators involved in lipid accumulation will help to elucidate the molecular mechanisms regulating lipid saturation and the complex regulatory networks of DHA synthesis in thraustochytrids.

## Conclusion

In conclusion, we constructed an engineered strain OPKSABC-PPT with high DHA productivity and DHA content through genetic engineering of the fatty acid biosynthetic pathways. The investigation also reveals the importance of the PKS and FAS pathways for growth and lipid accumulation of *Schizochytrium* sp. ATCC20888. This study demonstrates that genetic engineering of the fatty acid biosynthetic pathways provides an efficient strategy to enhance PUFA production in thraustochytrids.

## Materials and methods

### Strains and culture conditions

The *Schizochytrium* and *E. coli* strains used for the study are listed in Table S1. *Schizochytrium* sp. was grown and screened on solid GPY medium containing 2% glucose, 1% peptone, 0.5% yeast extract, 1% sea crystal, and 1.5% agar. And 50 μg/mL zeocin or 400 μg/mL G418 was used for selection of transformants. The medium for seed cultivation was composed of 3% glucose, 1% peptone, 0.5% yeast extract, 2% sea crystal. Shake-flask batch fermentations for production of lipid and DHA were performed in fermentation medium containing 10% glucose, 0.5% yeast extract, 0.59% NaCl, 0.026% KCl, 0.1% (NH_4_)_2_SO_4_, 0.1% KH_2_PO_4_, 0.143% MgSO_4_, and 0.004% CaCl_2_. *Schizochytrium* sp. was cultivated at 28°C and 230 rpm in seed medium for 24 h, and then, 5% (v/v) of cultures were transferred into fermentation medium. *E. coli* strain JM109 was cultivated at 37°C in LB medium supplemented with 50 μg/mL zeocin or 100 μg/mL kanamycin.

### Construction of mutants

The GenBank accession numbers for *fas*, *orfA*, *orfB*, *orfC*, and *ppt* are EF015632, AF378327, AF378328, AF378329, and PP026199 [5, 6]. Gene in situ overexpression mutant and in situ weakened mutant were constructed by homologous recombination strategy described previously [27]. For construction of *orfA* and *orfB* overexpression strain, the *ble* gene was amplified from plasmid pPICZaA (Invitrogen; Carlsbad, California, United States) with primers ble-o-Fw and ble-o-Rev as the selective marker and ligated with two *ccg1* promoters (amplified with primers ccg1p-up-Fw/ccg1p-up-Rev and ccg1p-dw-Fw/ccg1p-dw-Rev) flanking the *ble* gene in opposite directions (Table S2). The upstream and downstream homologous arms flanking the *orfA-orfB* intergenic region were amplified from *Schizochytrium* sp. ATCC20888 genomic DNA with primers orfA-up-o-Fw/orfA-up-o-Rev and orfB-dw-o-Fw/orfB-dw-o-Rev and ligated with *ccg1p*-*ble*-*ccg1p* by seamless cloning, respectively (Fig. S7). The resulting DNA fragments were electrically transformed jointly to ATCC20888, and overexpression mutants (OPKSAB) were selected on GPY medium with 50 μg/mL zeocin. For construction of the *fas*-weakened mutant, the *nat* gene was amplified from plasmid ptrpC-Nat [29] with primers nat-loxp-Fw/nat-loxp-Rev and ligated with weak promoter *4678p* (amplified with primers 4678p-Fw /4678p-Rev). The upstream and downstream homologous arms flanking the *fas* promoter region were amplified with primers fas-up-Fw/fas-up-Rev and fas-dw-Fw/fas-dw-Rev and ligated with *nat* or *nat*-*4678p* by seamless cloning, and together transformed to ATCC20888 to produce *fas*-weakened mutant WFAS (Fig. S1). For overexpression of *orfC* or *ppt*, the genes were amplified from the cDNA of ATCC20888 with primers orfC-Fw/orfC-Rev and ppt-Fw/ppt-Rev. The promoters and terminators of *ccg1* were amplified from the *Neurospora* expression vector pCCG.N-3xMyc with primers ccg1p-Fw/ccg1p-Rev and ccg1t-Fw/ccg1t-Rev; *EF1α* promoter was amplified from *Schizochytrium* sp. ATCC20888 with primers ef1αp-Fw/ef1αp-Rev; and the *CYC-1* terminator was amplified from pPICZαA with primers cyc1t-Fw/cyc1t-Rev. After purification, *EcoR*I/*Kpn*I-digested promoter fragments and *Kpn*I/*Xba*I-digested terminator fragments were ligated into *EcoR*I/*Xba*I-digested pPICZαA, respectively. The resulting plasmids were then digested with *Kpn*I and ligated to *orfC* or *ppt* using Seamless Cloning and Assembly Kit (Clone Smarter Technologies; USA) to produce overexpression plasmids (Tables S1; S2). The expression cassettes were amplified from the corresponding overexpression plasmids and together ligated to pPIC3.5 K (Invitrogen; Carlsbad, California, United States) by seamless cloning to produce co-overexpression plasmid. After linearization by *Pme*I, overexpression plasmids were transformed into *Schizochytrium* sp. WT or OPKSAB (Fig. S9). The parameters for electroporation are 1.5 kV, 4.5 ms, twice. After incubation at 28°C for 4 h in 1 mL of seed medium, the cells were plated on GPY media with appropriate antibiotics for transformants selection.

### Determination of biomass, glucose concentration, lipid yield and fatty acid composition

For biomass analysis, the cell pellets from 40 mL fermentation broth were collected by centrifugation (7000 g, 4°C, 5 min) and freeze-dried to constant weight. The glucose concentration of supernatant was detected by the 3,5-dinitrosalicylic acid method [30]. Lipid extraction and fatty acid methyl esters (FAMEs) preparation were carried out as described previously [31]. Briefly, approximately 200 mg of lyophilized *Schizochytrium* powder was resuspended in 6 mL of 4 M HCl and heated in a boil water bath for 30 min, and then 15 mL of methanol/chloroform (1:1, v/v) was added to extract the lipid. FAMEs were prepared from 50 mg lyophilized *Schizochytrium* powder and analyzed by gas chromatography as described previously [27].

### Thin layer chromatography (TLC) analysis of the extracted lipid

The lipid components in the samples were determined by TLC on silica gel plate using a mobile phase consisting of *n*-hexane: diethyl ether: acetic acid (85:15:1; v/v/v) [32]. The separated strips were photographed by spraying MnCl_2_-methanol solution (0.32 g MnCl_2_·4H_2_O, 30 mL methanol, 30 mL water, and 4 mL concentrated H_2_SO_4_) in a hot air oven at 85°C for 5 min. TAG and polar lipid (PL) strips were scraped from TLC plates and then methylated using the method described previously [31]. The SFAs and PUFAs components in TAG or PL were determined by TLC on silica gel plate using a mobile phase consisting of n-hexane: diethyl ether: acetic acid (94:4:2; v/v/v). The loading amount was adjusted to load similar amounts of SFAs or PUFAs between samples. Image J software was used to detect the gray value of each lane for statistics, and SFAs of WT was set as 1.

### Lipid detection and microscopic analysis by Nile red staining

The lipid production of strains can be detected directly and quickly by Nile red-based fluorescence assay [33]. *Schizochytrium* cells were grown in fermentation broth for 96 h. The harvested cells were collected by centrifugation (5000 g, 4°C, 5 min) and washed twice with PBS (38.7 mM Na_2_HPO_4_·12H_2_O, 11.3 mM NaH_2_PO_4_·2H_2_O, and 150 mM NaCl). Lipid was stained with Nile red dye (0.5 mg/L) in dark for 5 min. The mixtures were excited at wavelength of 488 nm and fluorescent emission was analyzed at 570 nm on a microplate reader (SpectraMax M5, Molecular devices, CA, USA). The *Schizochytrium* cells from 72-h-old culture were stained with Nile red dye and imaged using a LEICA TCS SP8 microscope equipped with an oil immersion objective (×1000 magnification) at 488 nm.

### Quantitative real-time PCR analysis (RT-qPCR)

Total RNA was extracted from 48 or 96 h-old *Schizochytrium* cells using TRIzol (Tiangen; China) according to the manufacturer’s protocol. cDNA was synthesized by M-MLV (RNase H^−^; TaKaRa Bio, Shiga, Japan) with oligo-dT18 as primers and 4 µg RNA as template. The FastStart Universal SYBR Green Master (ROX) was used for quantitative RT-PCR analysis of *orfA*, *orfB*, *orfC*, *fas* and *ppt* genes with primers listed in Table S2. The relative transcription level was calculated using the 2−ΔΔCt method, and actin was used as internal control.

### Supplementary Information

Supplementary material 1. Figure S1. Construction of WFAS. Figure S2. Phenotypes of WT, WFAS, and DPKSA. Figure S3. Confocal microscopy images of Nile red-stained cells grown in fermentation medium for 72 h. Figure S4. Schematic representation of insertion inactivation of *orfA*. Figure S5. Aerobic and anaerobic pathways for VLCPUFA biosynthesis in *Schizochytrium* sp. ATCC20888. Figure S6. Effects of decreased expression of *fas* or disruption of *orfA* on lipid accumulation. Figure S7. Construction of *orfAB* overexpression strain. Figure S8. Effect of enhanced fatty acid synthesis PKS pathway on lipid accumulation. Figure S9. PCR verification of overexpression mutants.

Supplementary material 2. Table S1. Strains and plasmids used in this study. Table S2. Primers used in this study.

## Data Availability

All data generated or analyzed during this study are included in this published article and its supplementary information files.

## Abbreviations

ACP: Acyl carrier protein
AT: Acyltransferase
CLF: Chain length factor
DCW: Dry cell weight
DH: Dehydratase
DHA: Docosahexaenoic acid
DPA: Docosapentaenoic acid
EPA: Eicosapentaenoic acid
ER: Enoyl-ACP reductase
FAMEs: Fatty acid methyl esters
FAS: Fatty acid synthase
KR: 3-Ketoacyl-ACP reductase
KS: 3-Ketoacyl-ACP synthase
MAT: Malonyl-CoA: ACP acyl transferase
PKS: Polyketide synthase
PPTase: Phosphopantetheinyl transferase
PL: Polar lipid
PUFAs: Polyunsaturated fatty acids
SFAs: Saturated fatty acids
TFAs: Total fatty acids
TLC: Thin layer chromatography
WT: Wild type
