# Supplementary material for: Selectively superior production of docosahexaenoic acid in Schizochytrium sp. through engineering the fatty acid biosynthetic pathways
Source: Biotechnol Biofuels Bioprod. 2024 Jun 3;17:75. doi: 10.1186/s13068-024-02524-2 (PMC11145866; doi:10.1186/s13068-024-02524-2)
Supplement: Supplementary file 1 — Supplementary material 1. Figure S1. Construction of WFAS. Figure S2. Phenotypes of WT, WFAS, and DPKSA. Figure S3. Confocal microscopy images of Nile red-stained cells grown in fermentation medium for 72 h. Figure S4. Schematic representation of insertion inactivation of orfA. Figure S5. Aerobic and anaerobic pathways for VLCPUFA biosynthesis in Schizochytrium sp. ATCC20888. Figure S6. Effects of decreased expression of fas or disruption of orfA on lipid accumulation. Figure S7. Construction of orfAB overexpression strain. Figure S8. Effect of enhanced fatty acid synthesis PKS pathway on lipid accumulation. Figure S9. PCR verification of overexpression mutants. [file 13068_2024_2524_MOESM1_ESM.pptx]

## Slide 1
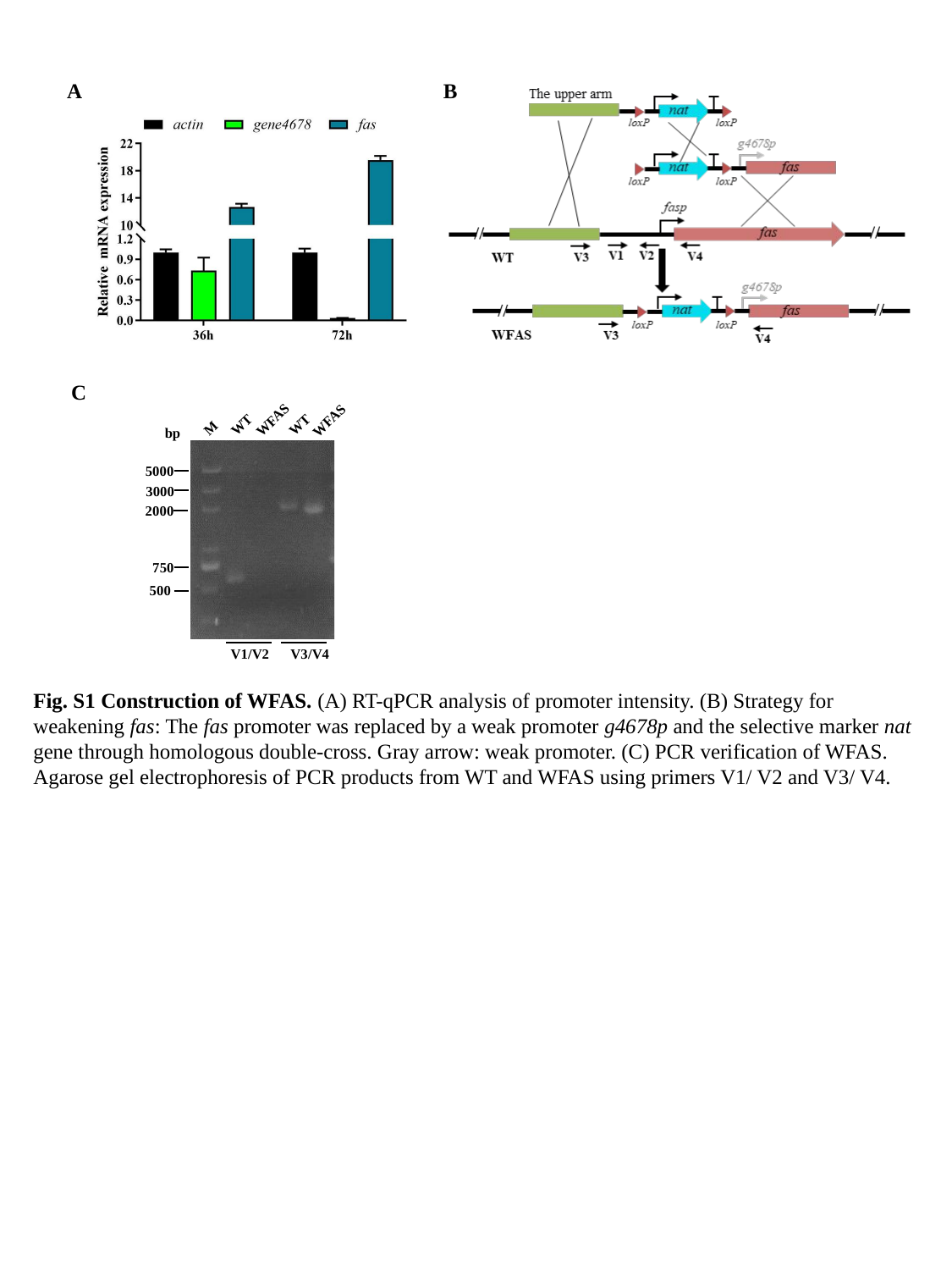

B
A
C
WFAS
WFAS
M
WT
WT
bp
5000
3000
750
500
V1/V2 V3/V4
2000
Fig. S1 Construction of WFAS. (A) RT-qPCR analysis of promoter intensity. (B) Strategy for weakening fas: The fas promoter was replaced by a weak promoter g4678p and the selective marker nat gene through homologous double-cross. Gray arrow: weak promoter. (C) PCR verification of WFAS. Agarose gel electrophoresis of PCR products from WT and WFAS using primers V1/ V2 and V3/ V4.

## Slide 2
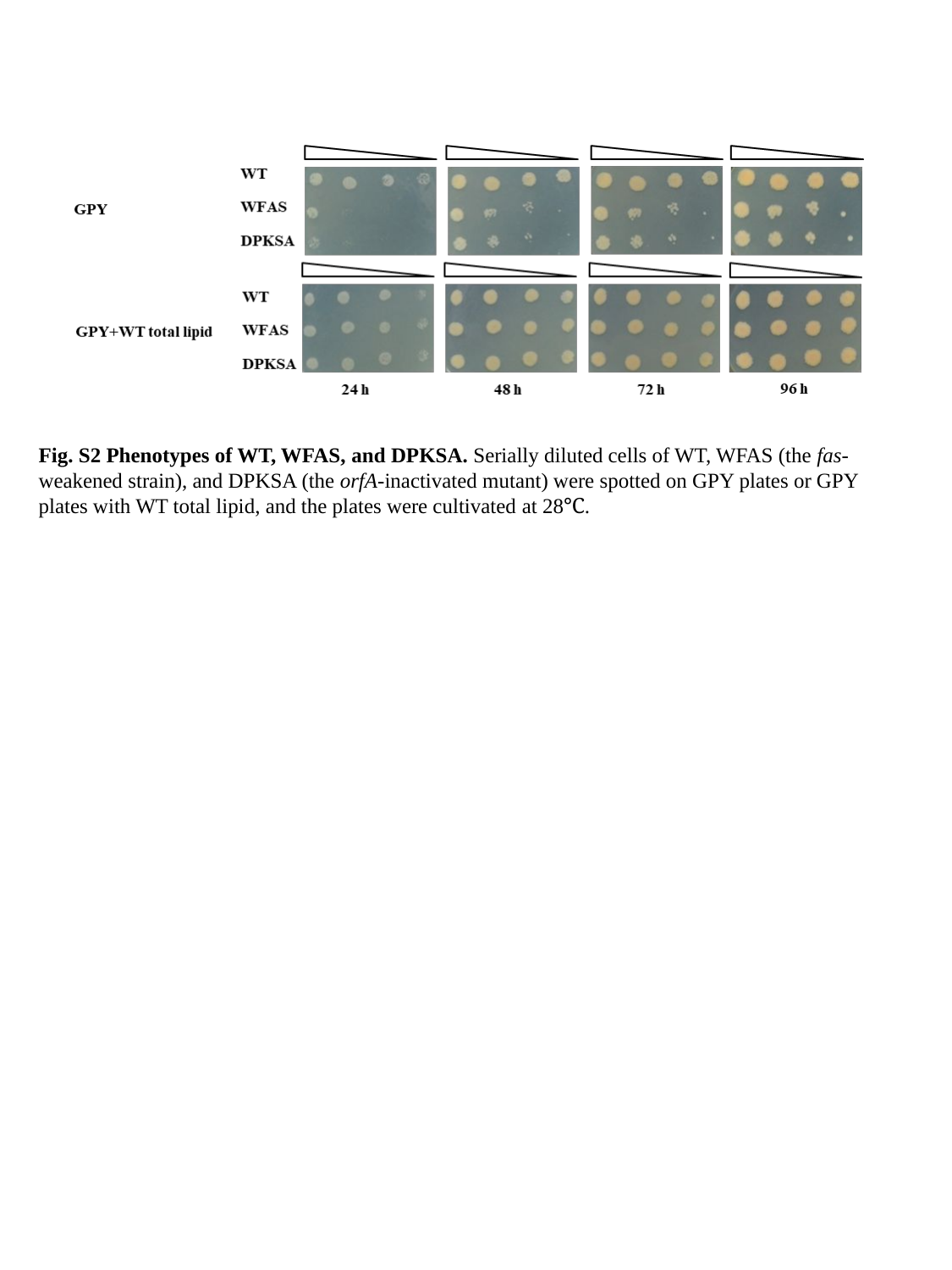

Fig. S2 Phenotypes of WT, WFAS, and DPKSA. Serially diluted cells of WT, WFAS (the fas-weakened strain), and DPKSA (the orfA-inactivated mutant) were spotted on GPY plates or GPY plates with WT total lipid, and the plates were cultivated ​at 28℃.

## Slide 3
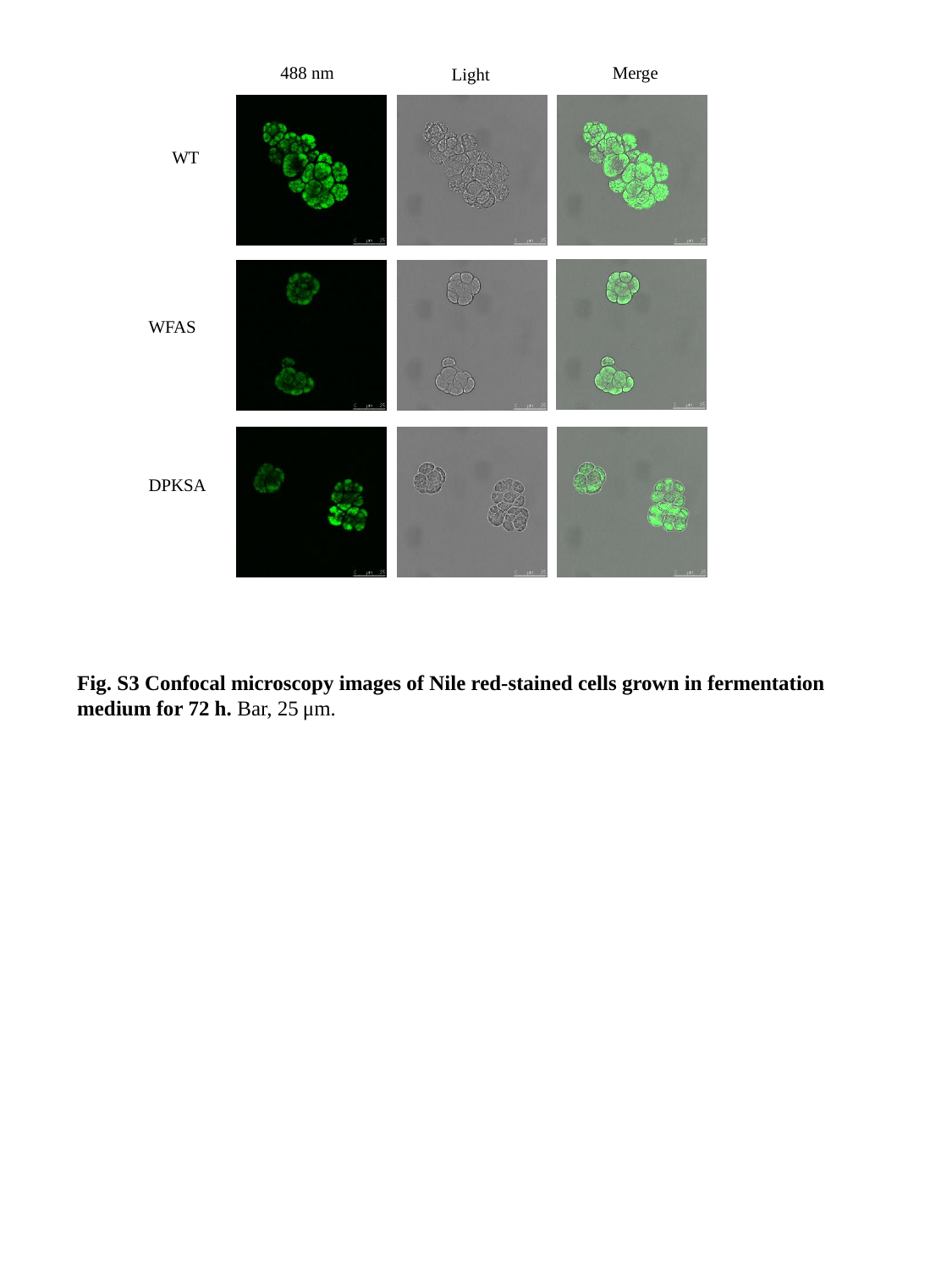

488 nm
Merge
Light
WT
WFAS
DPKSA
Fig. S3 Confocal microscopy images of Nile red-stained cells grown in fermentation medium for 72 h. Bar, 25 μm.

## Slide 4
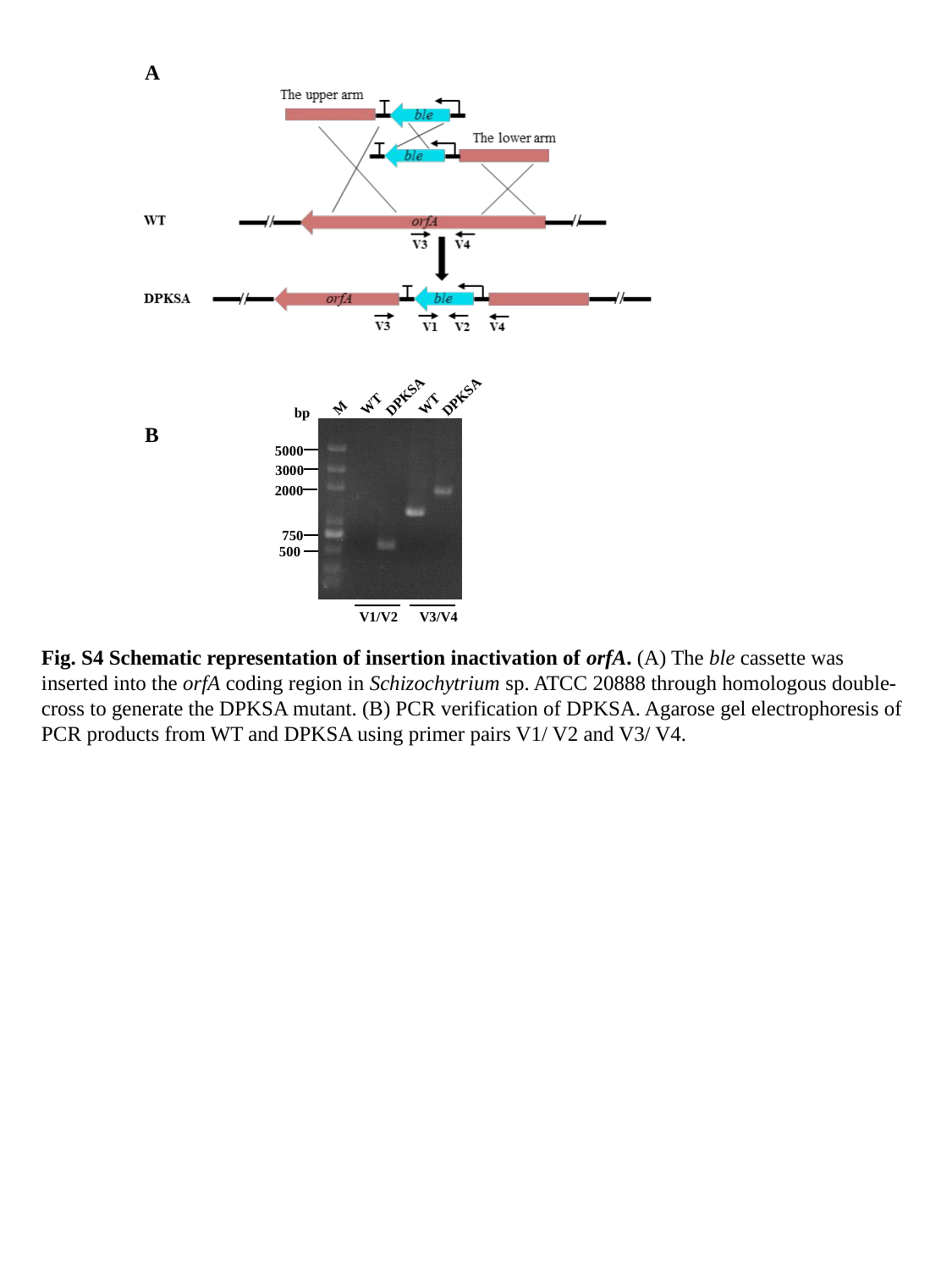

A
DPKSA
DPKSA
M
WT
WT
bp
5000
3000
750
500
V1/V2 V3/V4
2000
B
Fig. S4 Schematic representation of insertion inactivation of orfA. (A) The ble cassette was inserted into the orfA coding region in Schizochytrium sp. ATCC 20888 through homologous double-cross to generate the DPKSA mutant. (B) PCR verification of DPKSA. Agarose gel electrophoresis of PCR products from WT and DPKSA using primer pairs V1/ V2 and V3/ V4.

## Slide 5
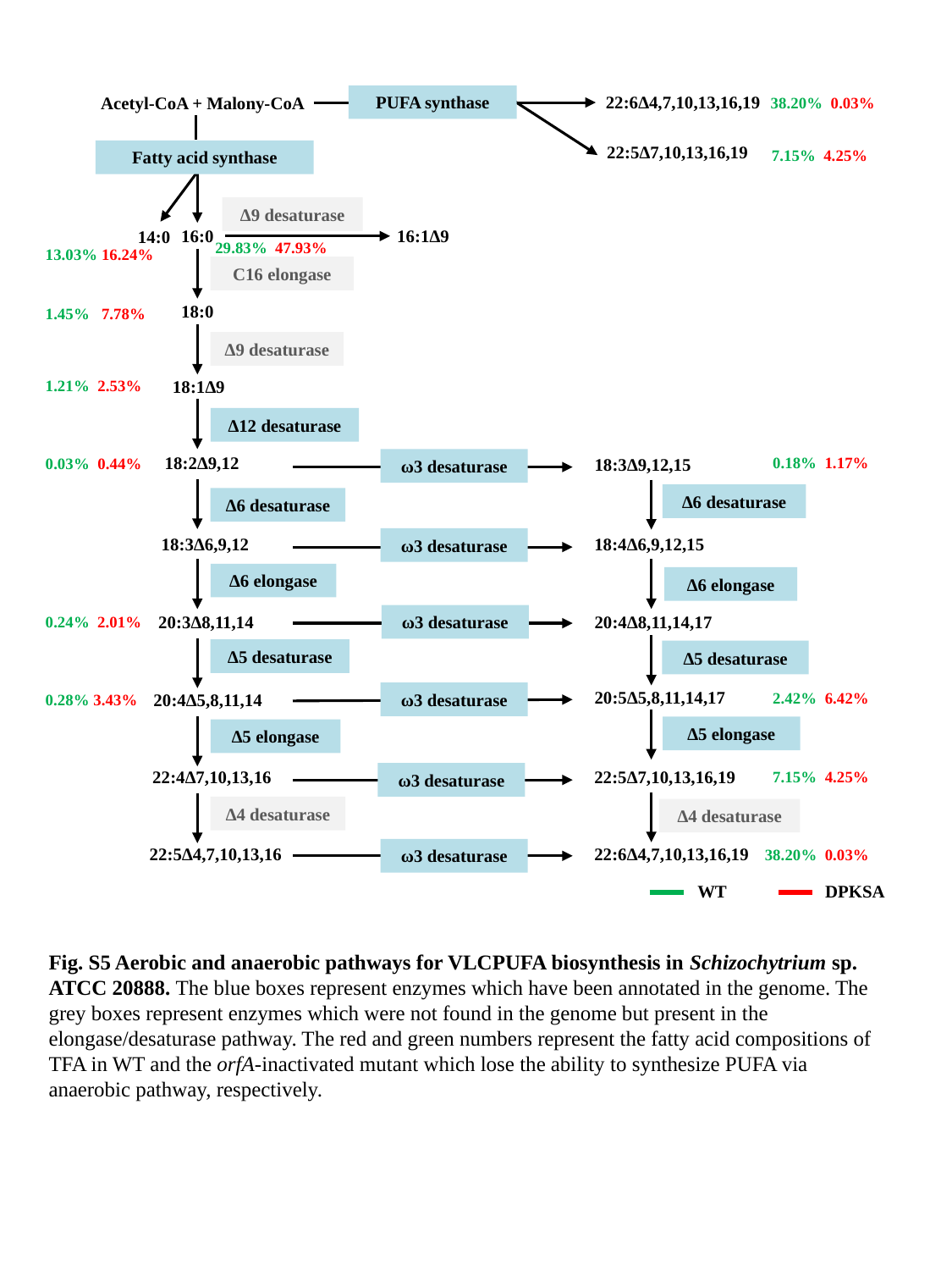

22:6∆4,7,10,13,16,19
PUFA synthase
Acetyl-CoA + Malony-CoA
38.20% 0.03%
22:5∆7,10,13,16,19
7.15% 4.25%
Fatty acid synthase
∆9 desaturase
16:0
16:1∆9
14:0
29.83% 47.93%
13.03% 16.24%
C16 elongase
18:0
1.45% 7.78%
∆9 desaturase
18:1∆9
1.21% 2.53%
∆12 desaturase
18:2∆9,12
0.18% 1.17%
18:3∆9,12,15
0.03% 0.44%
ω3 desaturase
∆6 desaturase
∆6 desaturase
18:3∆6,9,12
18:4∆6,9,12,15
ω3 desaturase
∆6 elongase
∆6 elongase
20:3∆8,11,14
20:4∆8,11,14,17
ω3 desaturase
0.24% 2.01%
∆5 desaturase
∆5 desaturase
20:5∆5,8,11,14,17
2.42% 6.42%
ω3 desaturase
20:4∆5,8,11,14
0.28% 3.43%
∆5 elongase
∆5 elongase
22:4∆7,10,13,16
22:5∆7,10,13,16,19
7.15% 4.25%
ω3 desaturase
∆4 desaturase
∆4 desaturase
22:5∆4,7,10,13,16
22:6∆4,7,10,13,16,19
38.20% 0.03%
ω3 desaturase
WT
DPKSA
Fig. S5 Aerobic and anaerobic pathways for VLCPUFA biosynthesis in Schizochytrium sp. ATCC 20888. The blue boxes represent enzymes which have been annotated in the genome. The grey boxes represent enzymes which were not found in the genome but present in the elongase/desaturase pathway. The red and green numbers represent the fatty acid compositions of TFA in WT and the orfA-inactivated mutant which lose the ability to synthesize PUFA via anaerobic pathway, respectively.

## Slide 6
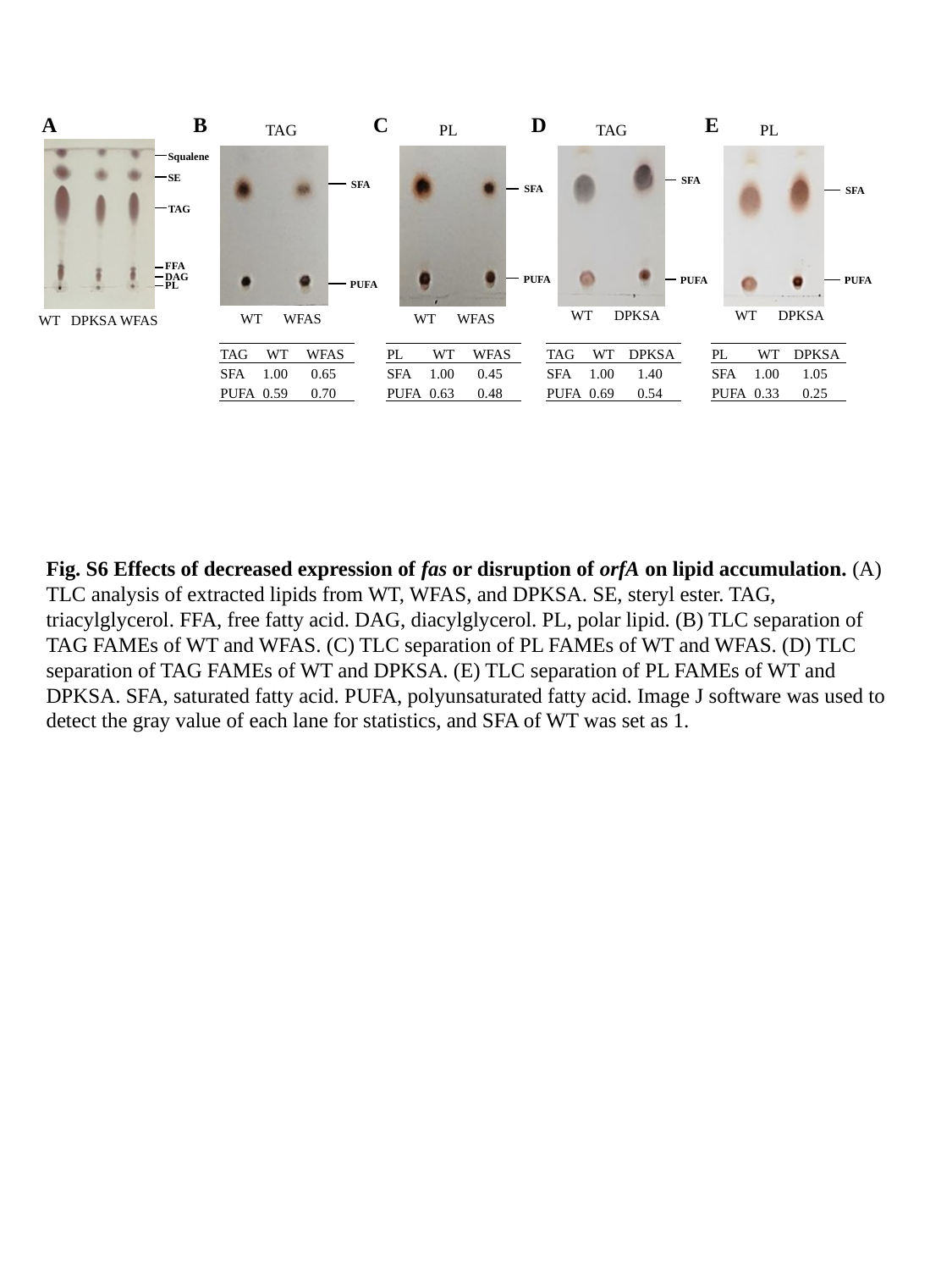

A
B
E
C
D
TAG
SFA
PUFA
WT WFAS
PL
SFA
PUFA
WT WFAS
TAG
SFA
PUFA
WT DPKSA
PL
SFA
PUFA
WT DPKSA
Squalene
SE
TAG
FFA
DAG
PL
WT DPKSA WFAS
| TAG | WT | WFAS |
| --- | --- | --- |
| SFA | 1.00 | 0.65 |
| PUFA | 0.59 | 0.70 |
| PL | WT | WFAS |
| --- | --- | --- |
| SFA | 1.00 | 0.45 |
| PUFA | 0.63 | 0.48 |
| TAG | WT | DPKSA |
| --- | --- | --- |
| SFA | 1.00 | 1.40 |
| PUFA | 0.69 | 0.54 |
| PL | WT | DPKSA |
| --- | --- | --- |
| SFA | 1.00 | 1.05 |
| PUFA | 0.33 | 0.25 |
Fig. S6 Effects of decreased expression of fas or disruption of orfA on lipid accumulation. (A) TLC analysis of extracted lipids from WT, WFAS, and DPKSA. SE, steryl ester. TAG, triacylglycerol. FFA, free fatty acid. DAG, diacylglycerol. PL, polar lipid. (B) TLC separation of TAG FAMEs of WT and WFAS. (C) TLC separation of PL FAMEs of WT and WFAS. (D) TLC separation of TAG FAMEs of WT and DPKSA. (E) TLC separation of PL FAMEs of WT and DPKSA. SFA, saturated fatty acid. PUFA, polyunsaturated fatty acid. Image J software was used to detect the gray value of each lane for statistics, and SFA of WT was set as 1.

## Slide 7
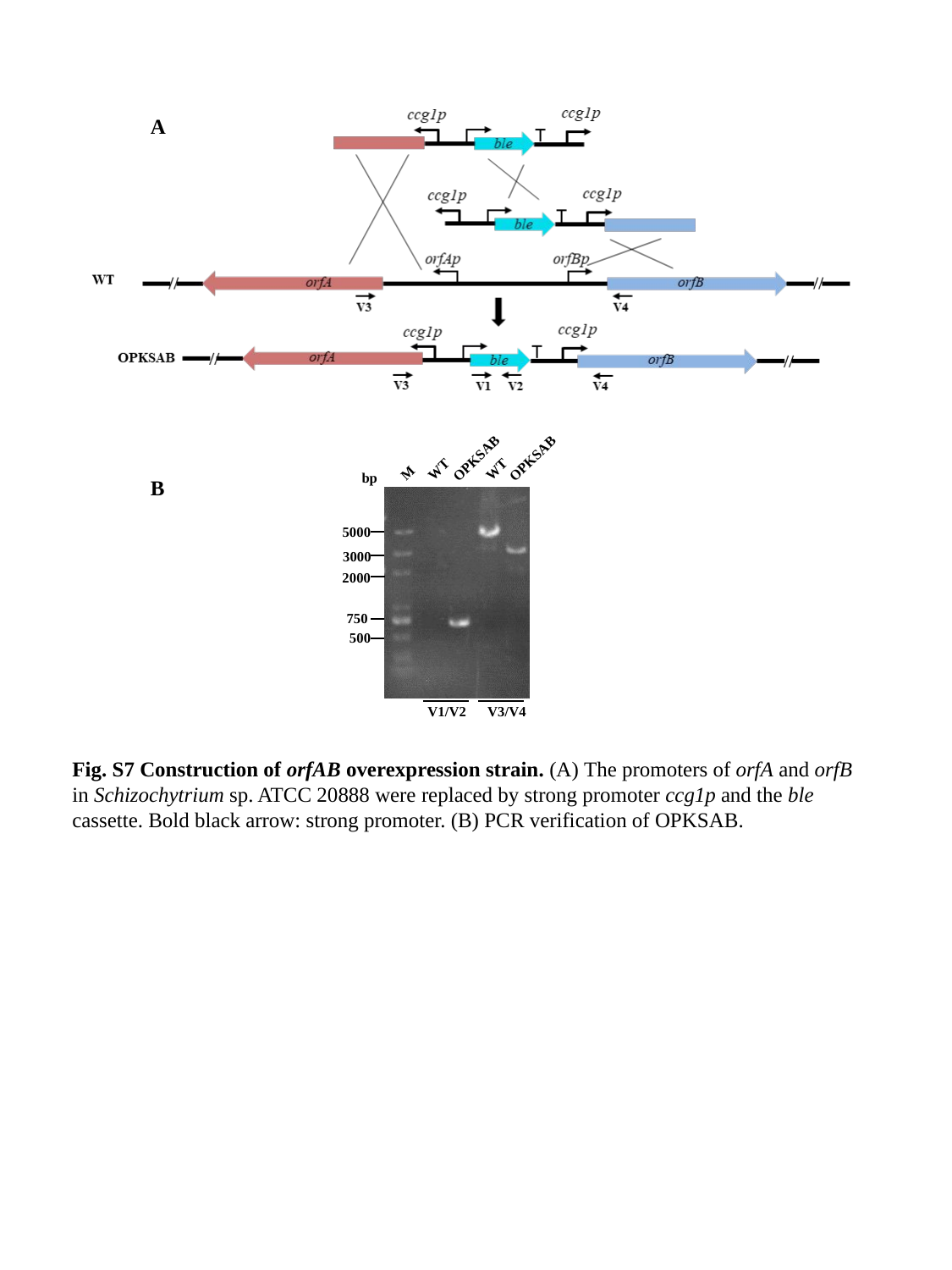

A
OPKSAB
OPKSAB
M
WT
WT
bp
5000
3000
750
500
V1/V2 V3/V4
2000
B
Fig. S7 Construction of orfAB overexpression strain. (A) The promoters of orfA and orfB in Schizochytrium sp. ATCC 20888 were replaced by strong promoter ccg1p and the ble cassette. Bold black arrow: strong promoter. (B) PCR verification of OPKSAB.

## Slide 8
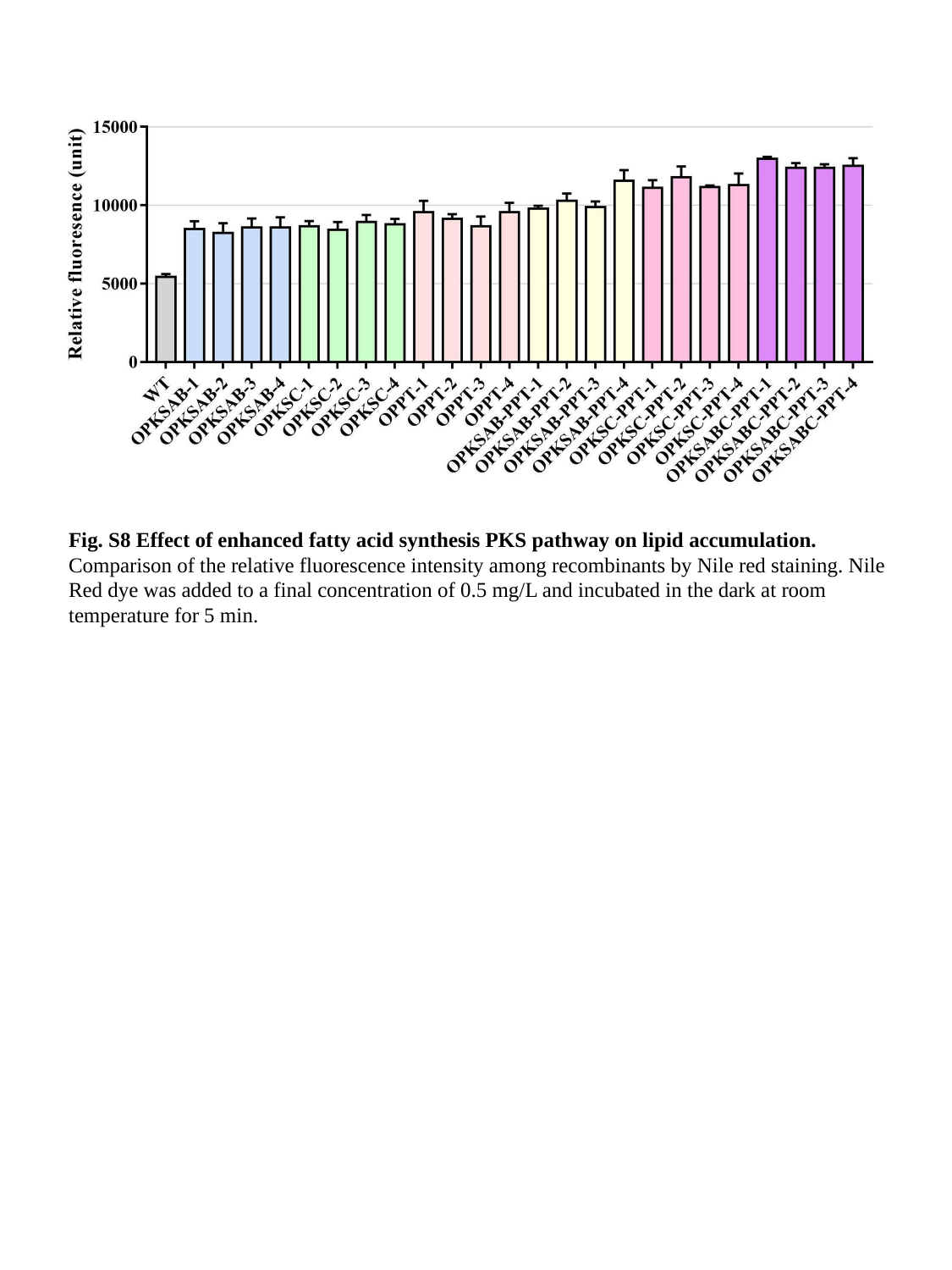

Fig. S8 Effect of enhanced fatty acid synthesis PKS pathway on lipid accumulation. Comparison of the relative fluorescence intensity among recombinants by Nile red staining. Nile Red dye was added to a final concentration of 0.5 mg/L and incubated in the dark at room temperature for 5 min.

## Slide 9
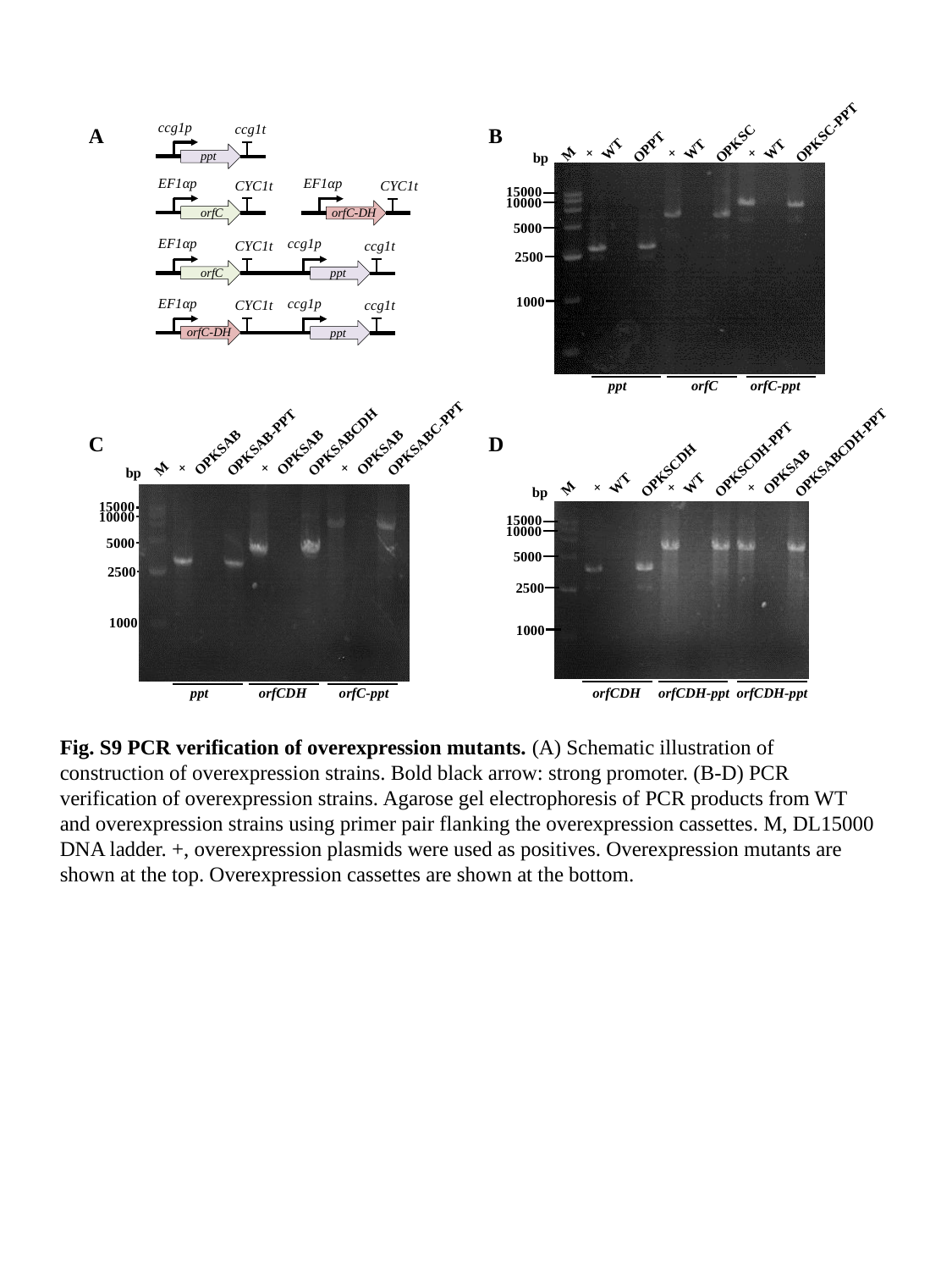

ccg1p
ccg1t
ppt
A
B
OPKSC-PPT
+
OPPT
WT
M
bp
15000
10000
5000
2500
1000
+
+
OPKSC
WT
WT
EF1αp
CYC1t
orfC
EF1αp
CYC1t
orfC-DH
EF1αp
CYC1t
orfC
ccg1p
ccg1t
ppt
EF1αp
CYC1t
ccg1p
ccg1t
ppt
orfC-DH
ppt orfC orfC-ppt
C
D
OPKSABC-PPT
OPKSABCDH
OPKSAB-PPT
OPKSAB
+
M
bp
15000
10000
5000
2500
1000
OPKSAB
OPKSAB
+
+
ppt orfCDH orfC-ppt
OPKSABCDH-PPT
OPKSAB
OPKSCDH-PPT
WT
WT
OPKSCDH
+
M
bp
15000
10000
5000
2500
1000
+
+
 orfCDH orfCDH-ppt orfCDH-ppt
Fig. S9 PCR verification of overexpression mutants. (A) Schematic illustration of construction of overexpression strains. Bold black arrow: strong promoter. (B-D) PCR verification of overexpression strains. Agarose gel electrophoresis of PCR products from WT and overexpression strains using primer pair flanking the overexpression cassettes. M, DL15000 DNA ladder. +, overexpression plasmids were used as positives. Overexpression mutants are shown at the top. Overexpression cassettes are shown at the bottom.
