# Supplementary material for: Selectively superior production of docosahexaenoic acid in Schizochytrium sp. through engineering the fatty acid biosynthetic pathways
Source: Biotechnol Biofuels Bioprod. 2024 Jun 3;17:75. doi: 10.1186/s13068-024-02524-2 (PMC11145866; doi:10.1186/s13068-024-02524-2)
Supplement: Supplementary file 2 — Supplementary material 2. Table S1. Strains and plasmids used in this study. Table S2. Primers used in this study. [file 13068_2024_2524_MOESM2_ESM.docx]

***Supplemental Materials***

Selectively superior production of docosahexaenoic acid in *Schizochytrium* sp. through engineering the fatty acid biosynthetic pathways

Yana Liu^a^, Xiao Han^a^, Zongcheng Chen^b^, Yihan Yan^a^, Zhi Chen^a^*

^a^ State Key Laboratory of Animal Biotech Breeding, College of Biological Sciences, China Agricultural University, Beijing 100193, China

^b^ College of Life Sciences, Qingdao Agricultural University, Qingdao 266109, China

*Corresponding author. Email: [chenzhi@cau.edu.cn](mailto:chenzhi@cau.edu.cn)

**Table S1. Strains and plasmids used in this study.**

| **Strain or plasmid** | **Description** | **Source or reference** |
| --- | --- | --- |
| ***Schizochytrium* sp.** |  |  |
| ATCC20888 | wild-type strain (WT) | American Type Culture Collection |
| DPKSA | *orfA*-inactivated mutant, *orfA*::*ble* | This study |
| WFAS | *fas*-weakened mutant, *fas* promoter was displaced by weak promoter *g4678p* | This study |
| OPKSAB | *orfA* and *orfB* co-overexpression strain, *orfA* and *orfB* promoters were displaced by strong promoter *ccg1p* | This study |
| OPKSC | *orfC* overexpression strain | This study |
| OPKSCDH | *orfC-DH* overexpression strain | This study |
| OPPT | *ppt* overexpression strain | This study |
| OPKSAB-PPT | *orfA*, *orfB*, and *ppt* co-overexpression strain | This study |
| OPKSC-PPT | *orfC* and *ppt* co-overexpression strain | This study |
| OPKSCDH-PPT | *orfC-DH* and *ppt* co-overexpression strain | This study |
| OPKSABCDH | *orfA, orfB*, and *orfC* *-DH* co-overexpression strain | This study |
| OPKSABCDH-PPT | *orfA, orfB*, *orfC-DH* and *ppt* co-overexpression strain | This study |
| OPKSABC-PPT | *orfA, orfB*, *orfC* and *ppt* co-overexpression strain | This study |
| ***E. coli*** |  |  |
| JM109 | General cloning host for plasmid manipulation | Laboratory stock |
| **Plasmids** |  |  |
| pPICZαA | Yeast expression vector | Invitrogen |
| pPIC3.5K | Yeast expression vector | Invitrogen |
| ptrpC-Nat | Vector carrying the *nat* gene | (He et al., 2020) |
| pPICZαA-PKSC | *orfC* overexpression plasmid based on pPICZαA | This study |
| pPICZαA-PKSCDH | *orfC-DH* overexpression plasmid based on pPICZαA | This study |
| pPICZαA-PPT | *ppt* overexpression plasmid based on pPICZαA | This study |
| pPIC3.5K-PPT-PKSCDH | *ppt* and *orfC-DH* co-overexpression plasmid based on pPIC3.5K | This study |
| pPIC3.5K-PPT-PKSC | *ppt* and *orfC* co-overexpression plasmid based on pPIC3.5K | This study |

**Table S2. Primers used in this study.**

| **Purpose** | **Primer** | **DNA sequence (5'–3')** | **Length (bp)** |
| --- | --- | --- | --- |
| For gene overexpression | | | |
| *EF1α* promoter | ef1αp-Fw | CCGGAATTCACTCGTCCCAGGGTGGTT (*Eco*RI) | 462 |
|  | ef1αp-Rev | GGGGTACCCAAAGTAGGCGGTAATAC (*Kpn*I) |  |
| *CYC-1* terminator | cyc1t-Fw | GGGGTACCAGCAGGACTGACACGTCC (*Kpn*I)) | 341 |
|  | cyc1t-Rev | GCTCTAGAATGTTGGTCTCCAGCTTGC (*Xba*I) |  |
| *ccg1* promoter | ccg1p-Fw | CCGGAATTCTCGTTCAAAGCCACATCA (*Eco*RI) | 854 |
|  | ccg1p-Rev | GGGGTACCTTGTTCCATGGATCCTTT (*Kpn*I) |  |
| *ccg1* terminator | ccg1t-Fw | GGGGTACCGCGCCATTAATTAACTAG (*Kpn*I) | 798 |
|  | ccg1t-Rev | GCTCTAGAAGCAAGAGCAACTAAACGG (*Xba*I) |  |
| *ppt* | ppt-Fw | CAAAGGATCCATGGAACAAGATGAGCACGGAGCAGCAG | 894 |
|  | ppt-Rev | ACTAGTTAATTAATGGCGCGTCAAGAAGATGCTTCTTT |  |
| *orfC* | orfC-Fw | GGTATTACCGCCTACTTTGGATGGCGCTCCGTGTCAAG | 4509 |
|  | orfC-Rev | CGGACGTGTCAGTCCTGCTGTTAGAGCGCGTTGGTGGG |  |
| *orfCDH* | orfCDH-Fw | GGTATTACCGCCTACTTTGGATGGCGCTCCGTGTCAAG | 2843 |
|  | orfCDH-Rev | CGGACGTGTCAGTCCTGCTGTTAGGCTGTCGGCCCAGAGGA |  |
| Construction of WFAS mutant | | | |
| *fas* upstream arm | fas-up-Fw | CAATGGAGACGGCCGAGGAT | 1360 |
|  | fas-up-Rev | GACAGTCGACCTGCAGCCCAATGGCGCAGCCCGAGTCG |  |
| *g4678* promoter | 4678p-Fw | CATTGGGCTGCAGGTCGACTGTCTCCTTTTTTCCAACTT | 982 |
|  | 4678p-Rev | CATACATTATACGAAGTTATTATCGGTTCAATTCGATCG |  |
| *nat* | nat-loxp-Fw | ATAACTTCGTATAATGTATGCTATACGAAGTTATCGGCATCTACTCTATTCC | 1021 |
|  | nat-loxp-Rev | ATAACTTCGTATAATGTATGCTATACGAAGTTATAGCTAGTGGAGGTCAACA |  |
| *fas* downstream arm | fas-dw-Fw | TGTATGCTATACGAAGTTATATGGACATCTCCGCTATCC | 1517 |
|  | fas-dw-Rev | GGAAGCATCTTGAACCTGTG |  |
| Construction of DPKSA mutant | | | |
| *orfA* upstream arm | orfA-up-d-Fw | TCGCAGCAATAGAGCTGC | 2118 |
|  | orfA-up-d-Rev | AAGGCTTTAATTTGCAAGCTTTGGCGAGGTTGTCGATGCC |  |
| *ble* | ble-d-Fw | GGCATCGACAACCTCGCCAAAGCTTGCAAATTAAAGCCTT | 1173 |
|  | ble-d-Rev | CCTCAGCCGCACCCGCACTGCCCACACACCATAGCTTCAA |  |
| *orfA* downstream arm | orfA-dw-d-Fw | TTGAAGCTATGGTGTGTGGGCAGTGCGGGTGCGGCTGAGG | 2134 |
|  | orfA-dw-d-Rev | TGCCAAGGAGGAGGCCAA |  |
| Construction of OPKSAB mutant | | | |
| *ccg1* promoter-1 | ccg1p-up-Fw | TCGTTCAAAGCCACATCA | 854 |
|  | ccg1p-up-Rev | TTGAAGCTATGGTGTGTGGGTTGTTCCATGGATCCTTT |  |
| *ble* | ble-o-Fw | AAAGGATCCATGGAACAACCCACACACCATAGCTTCAA | 1173 |
|  | ble-o-Rev | AGTGATGTGGCTTTGAACGAAGCTTGCAAATTAAAGCCTT |  |
| *ccg1* promoter-2 | ccg1p-dw-Fw | AAGGCTTTAATTTGCAAGCTTCGTTCAAAGCCACATCACT | 854 |
|  | ccg1p-dw-Rev | TTGTTCCATGGATCCTTT |  |
| *orfA* upstream arm | orfA-up-o-Fw | GATGGAGCCAGGGAATTTG | 1607 |
|  | orfA-up-o-Rev | AGGGAGGCGAGATGGATAC |  |
| *orfB* downstream arm | orfB-dw-o-Fw | ATGGCCGCTCGGAATGTG | 1573 |
|  | orfB-dw-o-Rev | CCTTGACGCCGCAGAGGT |  |
| For qRT-PCR | | | |
| *actin* | actin-QP-Fw | GCGACATCAAGGAGAAGC | 130 |
|  | actin-QP-Rev | GAAGGACGGCTGGAAGAG |  |
| *fas* | fas-QP-Fw | GCGACCAACAACACGGAC | 99 |
|  | fas-QP-Rev | CTGGGACTCCACAAATCC |  |
| *g4678* | g4678-QP-Fw | TGATGATGATAGTATGGCGG | 81 |
|  | g4678-QP- Rev | GTTGCTTTGGTTTCTTCCTC |  |
| *orfA* | orfA-QP-Fw | CGCCTCGGATTCACTTCG | 141 |
|  | orfA -QP-Rev | GCCCTGAGCAATGTCCAC |  |
| *orfB* | orfB -QP-Fw | GACATTCACCGCATTTGG | 134 |
|  | orfB -QP-Rev | AACTCTTGCTGCTGGCTC |  |
| *orfC* | orfC -QP-Fw | ACGATAACGACCACACCC | 179 |
|  | orfC -QP-Rev | GTCAGACACAGACACGGC |  |
| *ppt* | ppt-QP-Fw | AGTTCACCTTTCGTCTGTCAG | 131 |
|  | ppt-QP-Rev | ATCGTTCGTCTCAATGGC |  |

Underlining: Restriction endonuclease site.
